# Application of LDH assay for therapeutic efficacy evaluation of ex vivo tumor models

**DOI:** 10.1038/s41598-021-97894-0

**Published:** 2021-09-17

**Authors:** Megan C. Cox, Rita Mendes, Fernanda Silva, Teresa F. Mendes, Adelyn Zelaya-Lazo, Kathleen Halwachs, Julie J. Purkal, Inês A. Isidro, Ana Félix, Erwin R. Boghaert, Catarina Brito

**Affiliations:** 1 AbbVie, 1 North Waukegan Road, North Chicago, IL 60064-6098 USA; 2 IBET, Instituto de Biologia Experimental e Tecnológica, Apartado 12, 2780-901 Oeiras, Portugal; 3 Instituto de Tecnologia Química e Biológica António Xavier, Universidade Nova de Lisboa, Avenida da República, 2780-157 Oeiras, Portugal; 4 CEDOC-FCM-NOVA, Centro de Estudos de Doenças Crónicas da Faculdade de Ciências Médicas, Universidade Nova de Lisboa, R. Câmara Pestana 6, 1150-078 Lisbon, Portugal; 5 IPOLFG, Instituto Português de Oncologia de Lisboa Francisco Gentil, R. Prof. Lima Basto, 1099-023 Lisbon, Portugal; 6 The Discoveries Centre for Regenerative and Precision Medicine, Lisbon Campus, Av. da República, 2780-157 Oeiras, Portugal

**Keywords:** Cancer microenvironment, Cancer models, Drug development, Cancer models

## Abstract

The current standard preclinical oncology models are not able to fully recapitulate therapeutic targets and clinically relevant disease biology, evidenced by the 90% attrition rate of new therapies in clinical trials. Three-dimensional (3D) culture systems have the potential to enhance the relevance of preclinical models. However, the limitations of currently available cellular assays to accurately evaluate therapeutic efficacy in these models are hindering their widespread adoption. We assessed the compatibility of the lactate dehydrogenase (LDH) assay in 3D spheroid cultures against other commercially available readout methods. We developed a standardized protocol to apply the LDH assay to ex vivo cultures, considering the impact of culture growth dynamics. We show that accounting for growth rates and background release levels of LDH are sufficient to make the LDH assay a suitable methodology for longitudinal monitoring and endpoint assessment of therapeutic efficacy in both cell line-derived xenografts (xenospheres) and patient-derived explant cultures. This method has the added value of being non-destructive and not dependent on reagent penetration or manipulation of the parent material. The establishment of reliable readout methods for complex 3D culture systems will further the utility of these tumor models in preclinical and co-clinical drug development studies.

## Introduction

The current failure rate of new therapies in clinical trials is approximately 90%^1^. This high attrition rate, largely attributed to a lack of therapeutic efficacy, is a major factor driving the generation and implementation of clinically relevant models in drug discovery and development. The absence of such models has been suggested as a major reason why ineffective therapies reach clinical trials^2^. Because traditional preclinical model systems fail to reflect the complexity of human cancer, models that mimic the histiotypic tumor composition could represent greater physiological relevance than individually cultured cancer cells. In this regard, three-dimensional (3D) in vitro and ex vivo cultures may be more relevant to the clinic, while still maintaining the readability of reductionist approaches that study the cancer cell out of its physiological context.

The neoplastic tumor is composed of cancer cells comingled with a microenvironment containing an extracellular matrix (ECM), stromal cells, and a multitude of signaling factors capable of regulating therapeutic response across cancer types^3–6^. Microenvironmental components play a role in drug resistance and cancer progression^7^. Mechanisms of microenvironmentally-driven resistance include inhibition of drug tumor penetration, signaling pathway redundancy, and the presence of signaling factors that alter cancer cell behavior. Traditional monolayer screening platforms lack most microenvironmental components. Additionally, a complete understanding of the role tumor stroma plays in therapeutic efficacy is restricted by the limited capacity of standard in vitro model systems to only mimic the interactions between cancer cells and a select few components of the microenvironment^8^. Ex vivo models derived from xenografts or patient tumors incorporate the complexity of the parent tumor microenvironment into a preclinical system, namely tumor architecture and cancer cell-stromal cell interactions^9^.

Limitations to reliably assess therapeutic efficacy in these models by means of currently available cellular assays have so far impeded their widespread adoption in the drug development cascade. Standard readout methods include assays for cytotoxicity, proliferation, drug binding, apoptosis, and adenosine triphosphate (ATP) levels. Each come with their own inherent limitations that vary depending on cell type, cell number, and culture complexity. The simple addition of a 3D architecture can introduce reagent penetration limitations^10^. The incorporation of multiple cell types can further confound the determination of therapeutic efficacy by the inability of assays to distinguish between drug-induced cell death in cancer vs stromal cells^11^. It is therefore crucial to validate readout methods in complex culture formats, considering the advantages and limitations of each technique, and to create protocols for preclinical drug screening studies employing in vitro and ex vivo tumor models that are standardized, reproducible, and applicable to the culture system of choice^12^.

We have generated cultures from cell line-derived xenografts, termed xenospheres, and patient samples from surgical resection, termed patient-derived explants (PDE). The purpose of this study was to provide guidance on the application of the lactate dehydrogenase (LDH) assay for longitudinal monitoring and endpoint analysis of therapeutic efficacy in these complex culture models, where sample availability is highly limited. The LDH assay is suitable for longitudinal monitoring of cultures when employed to quantify the leakage of LDH from damaged cells to the culture supernatant. The assay does not require manipulation of the parent material, such as fluorescent labeling or gene transduction. It is not dependent on reagent penetration and, overall, is a non-destructive readout, making it compatible and highly attractive for PDE culture formats. The utility of the LDH assay was assessed in non-small cell lung cancer (NSCLC) 3D spheroid and xenospheres cultures against other longitudinal and non-longitudinal standard readout methods, including volume measurements, luminescence measurements of luciferase-expressing cells, and the CellTiter-Glo 3D (CTG-3D) assay. We then demonstrate that the LDH assay can assess the therapeutic efficacy of chemotherapeutics in ovarian cancer PDE (OvC-PDE) cultures.

## Results

### LDH assay reproducibility and precision is equivalent to other readouts commonly employed in 3D cell cultures

To determine whether the LDH assay can reliably assess cell growth and viability in 3D cultures, we compared the LDH assay to other established readout techniques commonly used to quantitatively measure cell viability in 3D cultures. Other criteria considered in the selection of readouts to directly compare the LDH assay include methods that are (1) compatible with low cell numbers due to the labor-intensive process of xenosphere generation and limited availability of patient material making methods requiring high cell numbers unfeasible in these culture formats; (2) do not require generation of single cell suspensions as the cell line and cell status may present distinct sensitivity to dissociation affecting assay results; (3) do not require sectioning (physical or optical) of the sample to capture the signal over the larger area of the xenospheres (~ 500–600 µm diameter) and PDE (1–2 mm diameter) which would involve a more complex experimental procedure and data processing.

**Figure 1:**
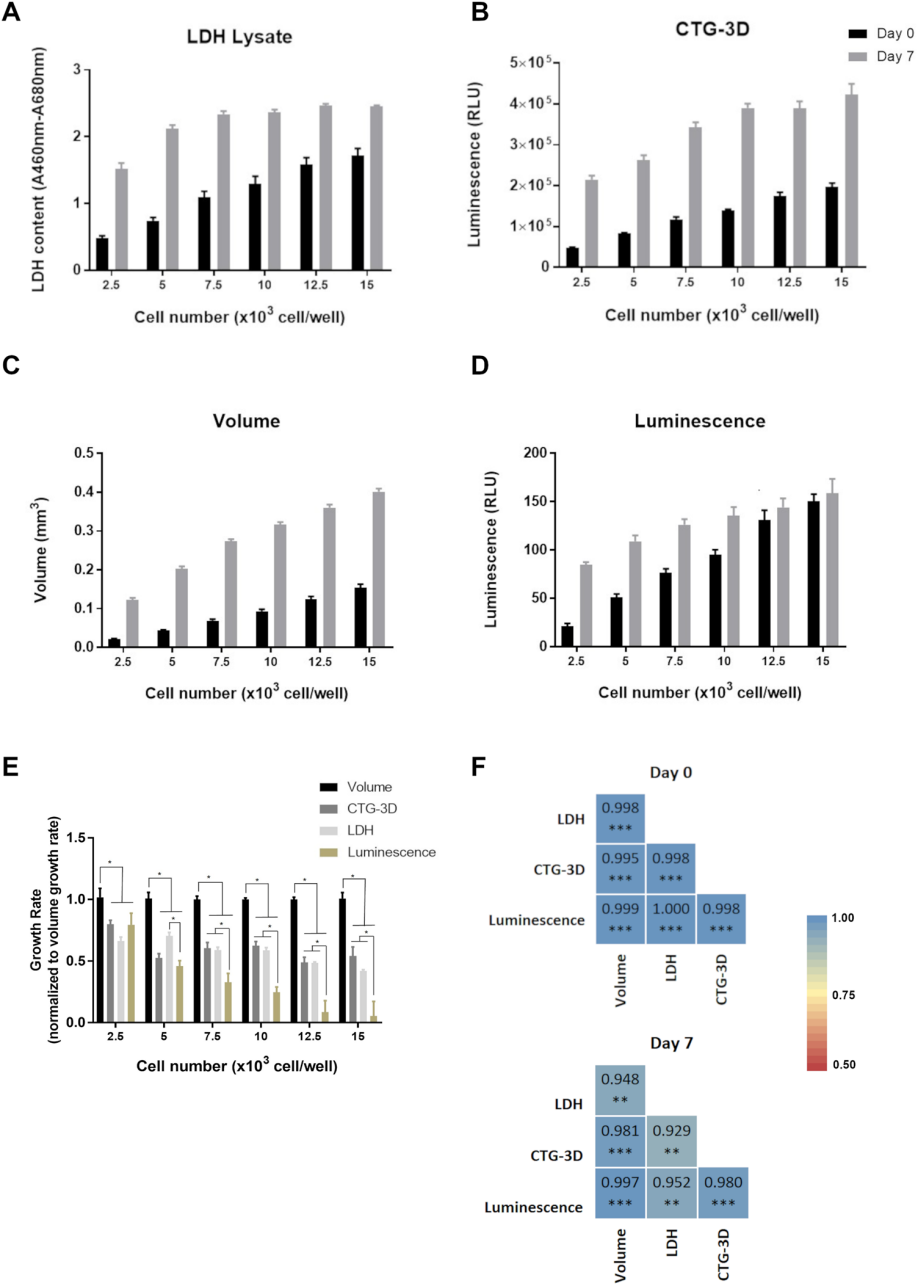
LDH assay reproducibility and precision in 3D culture is equivalent to alternate readout methods. (**A**) Lactate dehydrogenase (LDH) content, (**B**) CellTiter-Glo 3D (CTG-3D), (**C**) volume, and (**D**) luminescence measurements were collected from NCI-H1650 and/or NCI-H1650.LMC spheroids generated by seeding 2500, 5000, 7500, 10,000, and 12,500 cells per well of ultra-low attachment round bottom plates. LDH content and volume measurements were collected from both NCI-H1650 and NCI-H1650.LMC spheroids, CTG-3D readings were collected from NCI-H1650 spheroids only, and luminescence readings were collected from NCI-H1650.LMC spheroids only. Statistics for data presented in (**A**–**D**) is summarized in Supplementary Tables 1–3. (**E**) Growth rates for each seeding density condition calculated from cell content determined by each readout method was normalized to the growth rate determined from volume measurements. (**F**) Pearson correlation coefficient between volume and LDH, CTG-3D, and luminescence measurements (data are presented as mean ± SD of N = 5, *p < 0.05, **p < 0.01, ***p < 0.001).

We used spheroids of NCI-H1650 cells, as their generation is highly reproducible and a bioluminescent variant of the cells (NCI-H1650.LMC) is available, making the culture particularly suited to our purpose. NCI-H1650 and NCI-H1650.LMC spheroids, generated by seeding 2500, 5000, 7500, 10,000, 12,500, and 15,000 cells in ultra-low attachment round bottom plates, were evaluated via volume, CTG-3D, LDH, and luminescence, on day 0 and 7 of culture (Fig. 1). PrestoBlue and MTS assays were also evaluated but reagent penetration limitations resulted in their elimination as suitable options for universal assessment of viability in 3D cultures (Supplementary Fig. 1A, B). In contrast, discernment between seeding densities was not limited by reagent penetration in the LDH and CTG-3D assays (Supplementary Fig. 1C, D). Each readout could measure 5000-cell differences between seeding density on day 0, and each readout detected cell growth from day 0 to 7, except for luminescence that did not detect significant differences between day 0 and day 7 for seeding densities of 12,500 and 15,000 cells per well (Fig. 1A–E and Supplementary Tables 1–3). This might indicate a limitation of the luminescence readout to accurately report cell content as culture size increases, which may be due to diffusion limitations. In LDH measurements, saturation occurred at higher cell densities on day 7, but this could be overcome in future assays by diluting samples prior to analysis. The most sensitive readout to determine cell growth was volume, which together with LDH and CTG-3D, detected differences in cell content across all seeding densities (Fig. 1A–E and Supplementary Tables 1–3). However, the comparison of data sets via Pearson correlation analysis showed a strong correlation (*R*^2^ > 0.9) between volume measurements and all other readout methods, including LDH (Fig. 1F).

### Culture dynamics alter LDH assay data analysis

**Figure 2:**
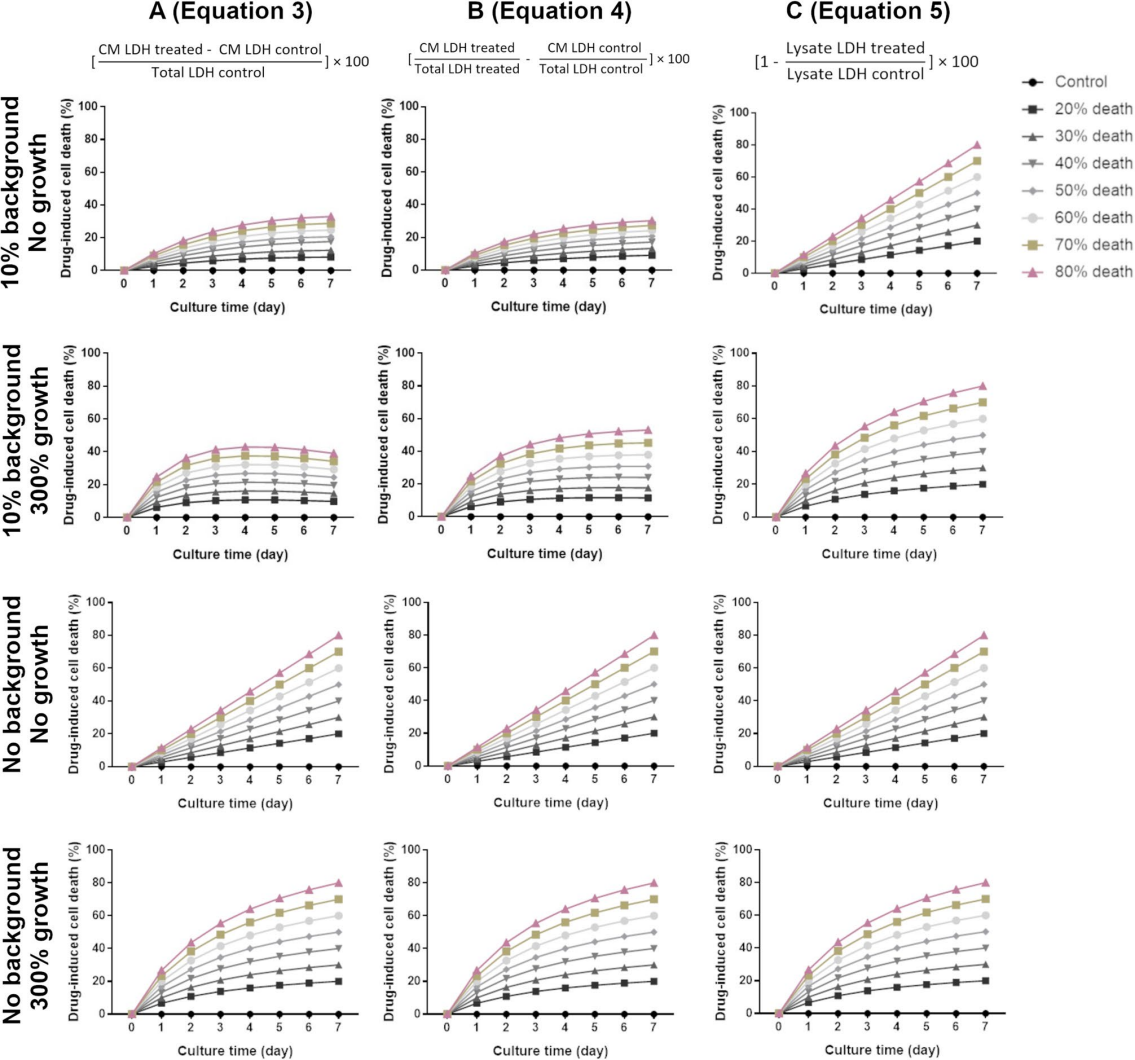
Culture growth dynamics impact how LDH data should be analyzed to evaluate therapeutic efficacy. Simulations represent cases in which a therapy that induces 0–80% cell death over the 7-day culture period has been applied to cultures that have 10% or no background LDH and do not grow or cell number triples over the culture period. Drug-induced cell death was calculated employing Eq. (3) (**A**), Eq. (4) (**B**) or Eq. (5) (**C**).

Longitudinal monitoring is a significant benefit of the LDH assay. However, differences in culture growth dynamics, including growth rate, death rate, and background LDH release in control and treated samples, may impact assessments of therapeutic efficacy. LDH data was simulated to ascertain how these factors might affect the determination of cell death (Fig. 2). Simulations represent drug-induced cell death calculated from LDH measurements in cases of no cell growth or cell tripling over the culture period (300% cell growth) and the presence of no background release or 10% background release, during treatment that induces 0–80% cell death over a 7-day culture period. Generally, the calculation of drug-induced death by LDH assay takes into consideration the amount of LDH that leaked to the culture medium in the treated group (conditioned medium, CM) and assumes the total cell content is equal in the treated and control groups (Eq. 3). The LDH content of the cell lysate can then be determined only for a control group and the percentage of drug-induced cell death defined as in Eq. (3). This assumption allows for the determination of cell death during longitudinal measurements at intermediate timepoints without sacrificing treated group samples. However, as shown in Fig. 2A,B, calculations based on CM LDH readings (Eqs. 3 and 4) are most accurate if there is no background LDH released by the cells. In cases where background release occurs, drug-induced cell death is underestimated as LDH content in the CM of the control sample will continue to increase throughout the culture, resulting in a disproportionate comparison of LDH content in the CM of treated to control samples (Fig. 2A,B). Thus, the determination of drug-induced cell death based on LDH content of the cell lysate (Eq. 5, Fig. 2C) is most accurate, as it considers the LDH content of live cells present in the treated and control group, and background release is not considered. For the selected therapeutic efficacy range, culture growth dynamics do not appear to impact the determination of cell death using any analysis method. However, for higher death rates (Supplementary Fig. 2A), Eqs. (3) and (4) can again underestimate cell death. In this case, frequent longitudinal measurements are necessary for accurate stack ranking using Eqs. (3) and (4).

### LDH assay can effectively assess standard-of-care (SOC) efficacy in xenosphere cultures

**Figure 3:**
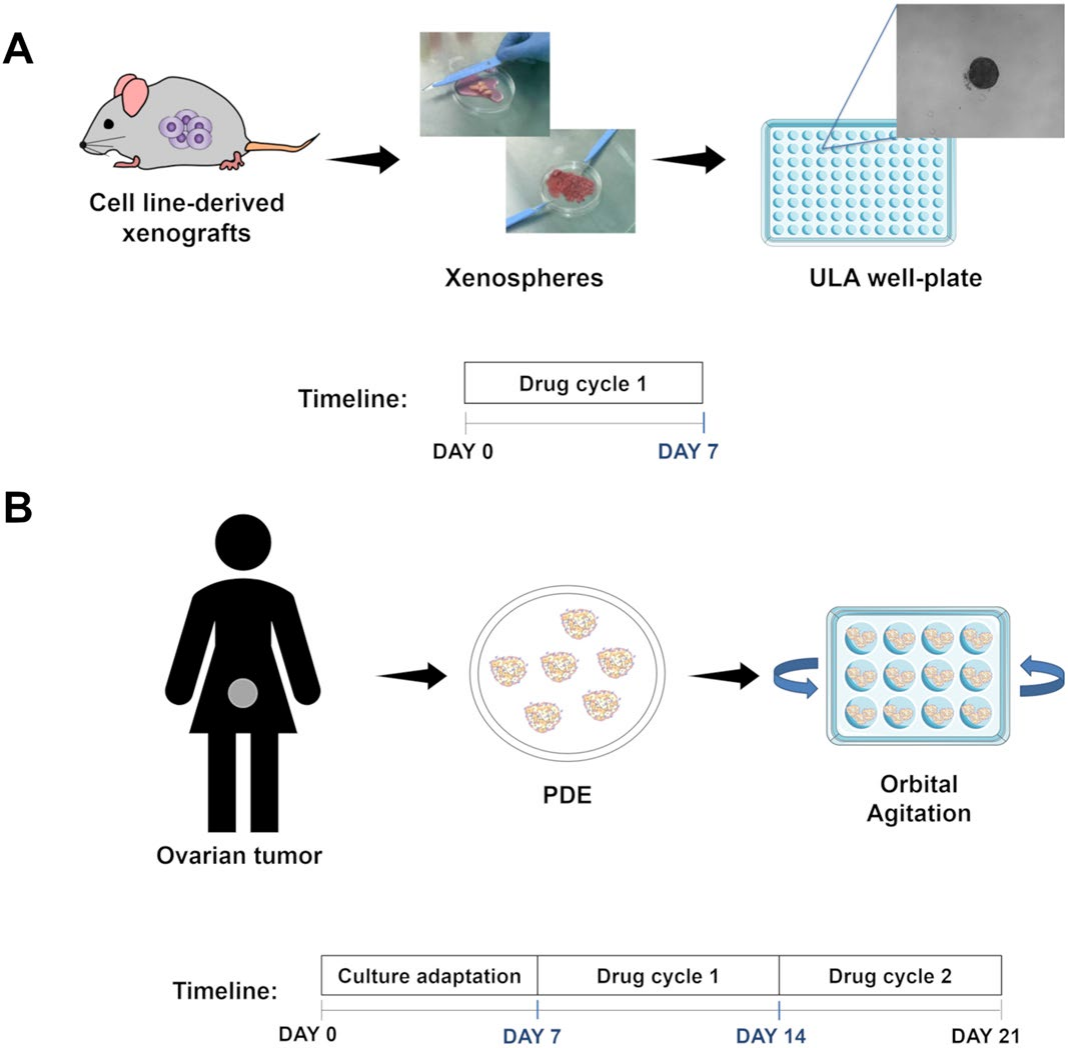
Schematic representation of the culture generation and drug cycle strategy pursued for ex vivo cultures. (**A**) Mouse xenografts are mechanically dissociated to form xenosphere explants. Individual xenospheres are cultured in a single well of a 96-well ultra-low attachment (ULA) plate. Docetaxel (DTX) treatments of 0.1, 5, 25, 125, 625, and 3125 nM are added directly to the xenospheres on the day of culture generation. Cell death in xenosphere cultures is assessed on day 7 by LDH assay. (**B**) Surgical-resected ovarian tumor specimens are mechanically dissociated into fragments, named patient-derived explants (PDE) and cultured in 12-well plates at a concentration of 5 PDE/ml, under orbital agitation at 100 rpm. After a 7-day adaptation to culture period, PDE cultures are challenged weekly with standard-of-care (SOC) chemotherapy, namely carboplatin (25 mg/ml) or paclitaxel (10 mg/ml) as single agents or in combination. Cell death is assessed longitudinally (day 7, 14, and 21 of culture) by LDH assay.

**Figure 4:**
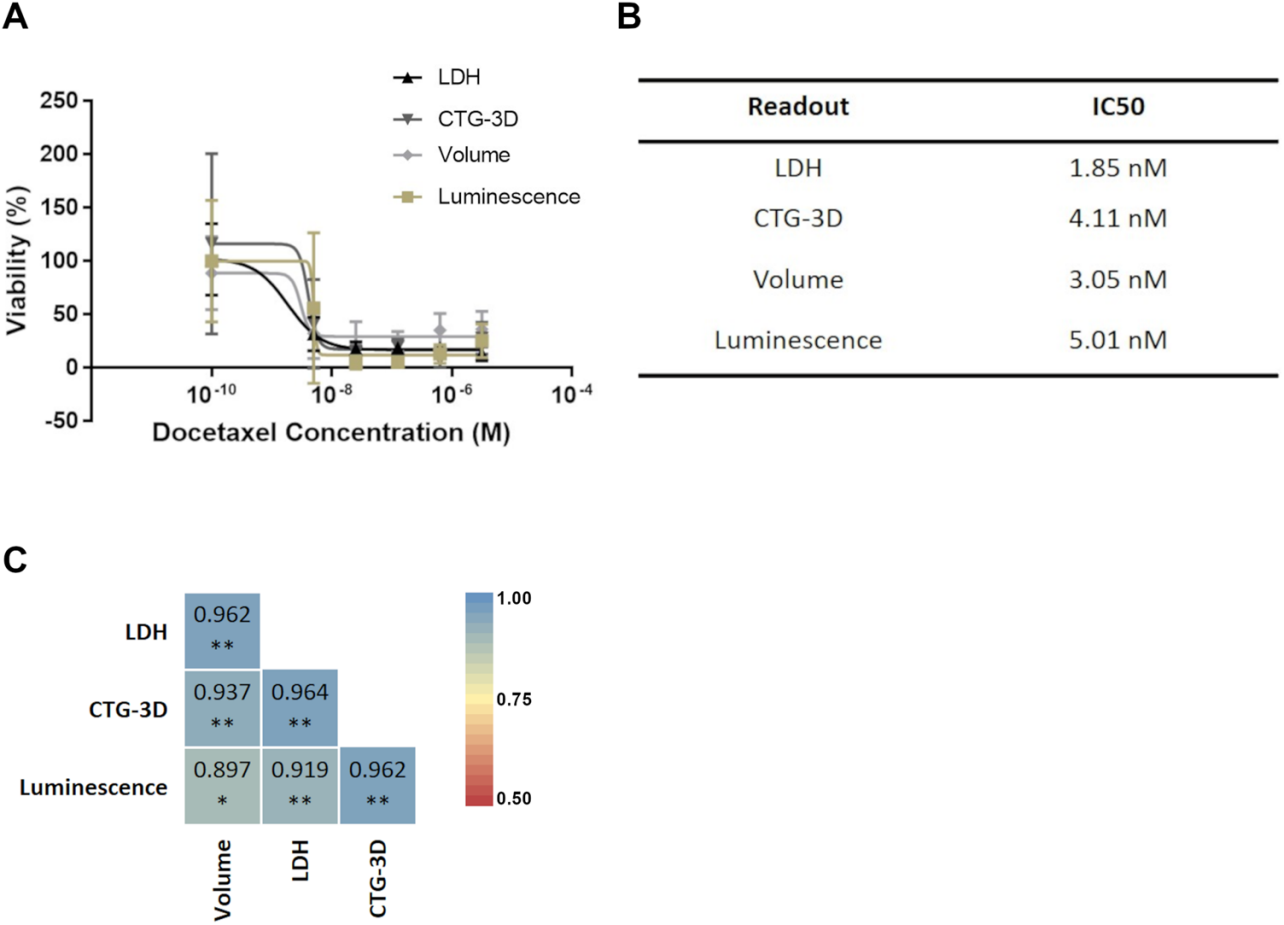
LDH assay can be used to assess DTX efficacy in xenosphere cultures. (**A**) DTX efficacy in xenosphere cultures was assessed via LDH, CTG-3D, and volume in NCI-H1650 xenospheres or luminescence in NCI-H1650.LMC xenospheres. (**B**) IC50 values of DTX in the xenosphere cultures as determined by each readout method, (**C**) Pearson correlation coefficient between measured LDH content in the cultures after DTX treatment and luminescence, CTG-3D, and volume measurements (data is presented as mean ± SD of N = 5, *p < 0.05 **p < 0.01).

Xenospheres were generated from xenograft tumors grown in mice. The generation of these cultures is fully described in the “Methods” section and illustrated in Fig. 3. Characterization by hematoxylin and eosin (H&E) is presented in Supplementary Fig. 3. We treated NCI-H1650 and NCI-H1650.LMC xenospheres with 0.1, 5, 25, 125, 625, and 3125 nM of the SOC, Docetaxel (DTX). Culture viability was assessed via LDH assay and compared with volume, CTG-3D, and luminescence measurements (Fig. 4A). For the LDH assay in Fig. 4A, culture viability was determined following Eq. (5), as the xenospheres cultures release 8.5% ± 2.8 of background LDH and grow over the 7-day culture period (Supplementary Fig. 2Bi). Utilizing Eqs. (3) or (4) to analyze the LDH assay data would underestimate the drug-induced cell death due to the dynamics of the xenosphere culture (Supplementary Fig. 4). The IC50 values, as determined by each readout method, were within the same order of magnitude (Fig. 4B). Pearson correlation analysis showed a strong correlation (*R*^2^ > 0.9) between the LDH assay and all other readout methods (Fig. 4C).

### Variations in OvC-PDE response to SOC chemotherapy captured by LDH assay

OvC patients usually undergo surgery followed by adjuvant chemotherapy, consisting of a combination of carboplatin and paclitaxel (C + P). Sixteen OvC samples from different subtypes were enrolled in this study and processed as described in the “Methods” section and illustrated in Fig. 3. Clinical-pathological parameters related to these tumors are reported in detail in Supplementary Tables 7 and 8. We previously developed an OvC-PDE model^13^ in which tumor architecture and cell type heterogeneity are preserved for at least 1 month in culture. This culture strategy can be broadly applied for the culture of different OvC types^13^. Here, we downscale this model to allow for simultaneous efficacy testing of multiple drugs. The 10 PDE/well scale was selected since cell viability was maintained after the 7-day adaption to culture period, and tumor architecture, cell phenotype, proliferation and apoptosis levels of the original tumor were also retained throughout 21 days in culture, similar to what we have previously described^13^ (Supplementary Figs. 5A,B and 6 and Supplementary Table 9).

**Figure 5:**
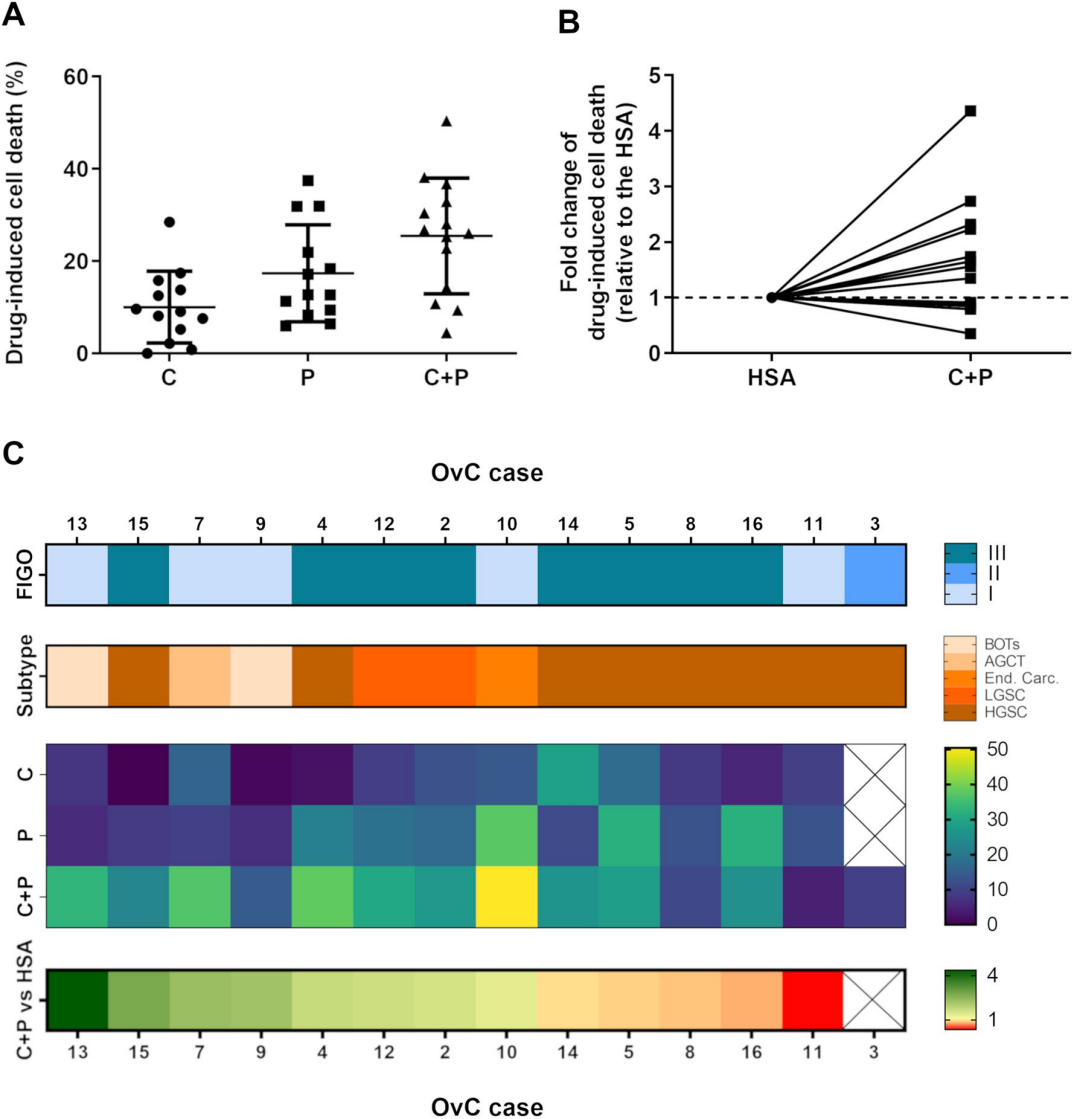
LDH assay can be used to assess SOC chemotherapy and single agent efficacy in PDE. (**A**) Drug-induced cell death evaluation of OvC-PDE using LDH assay after 2 drug cycles (data is presented as mean ± SD of N = 13–14). (**B**) Fold change of the combination drug-induced cell death relative to the drug-induced cell death of the highest single agent (HSA), i.e., carboplatin for OvC7, OvC13 and OvC14 and paclitaxel for all the other OvC cases (N = 13). (**C**) Patient sample-specific differences in drug response and combination effects is captured in the heatmap (organized by highest to lowest fold change of C + P-induced cell death relative to the HSA). (OvC: ovarian cancer; C: carboplatin; P: paclitaxel; BOTs: Borderline Ovarian Tumors; AGTC: Adult Granulosa Cell Tumor; End. Carc: Endometrioid Carcinoma; LGSC: Low-grade Serous Carcinoma; HGSC: High-grade Serous Carcinoma).

We asked if patient-specific and time-dependent variations in drug response would be captured by the LDH assay in a setup of long-term drug challenge of OvC-PDE cultures, with repeated evaluation of the same culture well over several cycles of chemotherapy. We challenged OvC-PDE cultures derived from 13 patients with two cycles of SOC or single-agent chemotherapy, over 2 weeks of culture (Fig. 3). Drug-induced cell death was evaluated by the LDH assay after each cycle of therapy. Having established that OvC-PDE did not grow over the culture period (Supplementary Fig. 2Bii), the drug-induced cell death could be calculated by an adaptation of Eq. (3), in which the two cycles of chemotherapy are taking into account (Eq. 6, as described in the “Methods” section). This implied that only control conditions needed to be lysed, so the explants exposed to the drugs could be observed over time, after the first and second drug cycles. Therefore, we could rank SOC and single agent efficacy among OvC-PDE derived from different patients, while preserving the biological material. For most OvC-PDE cultures, the first drug cycle was more effective than the second (Supplementary Fig. 7). The mean drug-induced cell death after two drug cycles, and its variability, changes with each treatment (Fig. 5A). Carboplatin induced the lowest response in terms of cell death (10.0% ± 7.8), followed by paclitaxel (17.4% ± 10.5) and the SOC chemotherapy (25.5% ± 12.6). The greatest response variation was observed after SOC chemotherapy treatment (Fig. 5A,C).

To further analyze the effect of combination therapy, we compared the combination effect (C + P) with the most efficacious single agent, henceforth referred to as the highest single agent (HSA)^14^ (Fig. 5B,C). In OvC7, OvC13, and OvC14, the HSA was carboplatin, whereas for the other cases, it was paclitaxel (Fig. 5B,C). We observed a negative combination effect (fold change, FC < 1), meaning the combination was less efficacious than the HSA, in 4 out of 13 (31%) OvC-PDE cases. These four cases correspond to samples derived from high-grade serous carcinoma (HGSC, OvC5, OvC8, OvC11 and OvC16). Additionally, OvC11 and OvC8 were the overall lowest responders to the combination treatment, with 4.5% ± 2.0 and 10.8% ± 0.8 drug-induced cell death, respectively (Fig. 5B,C and Supplementary Fig. 7). In one (8%) case (OvC14), the drug combination was as effective as the HSA (FC = 1). In the remaining eight (61%) cases, we observed a positive combination effect (FC > 1), meaning the combination was more efficacious than the HSA. OvC2, OvC4, OvC10, and OvC12 presented a FC ranging from 1.3 to 1.7 and were derived from HGSC, low-grade serous carcinoma (LGSC) and endometrioid carcinoma samples. The remaining four cases (OvC7, OvC9, OvC13 and OvC15) had a FC greater than 2 and were derived from sex-cord stromal, borderline ovarian tumors and HGSC (Fig. 5C). No correlation was observed between the drug combination-induced cell death and the FC of the combination to the HSA (Pearson correlation of *R*^2^ = 0.3).

**Figure 6:**
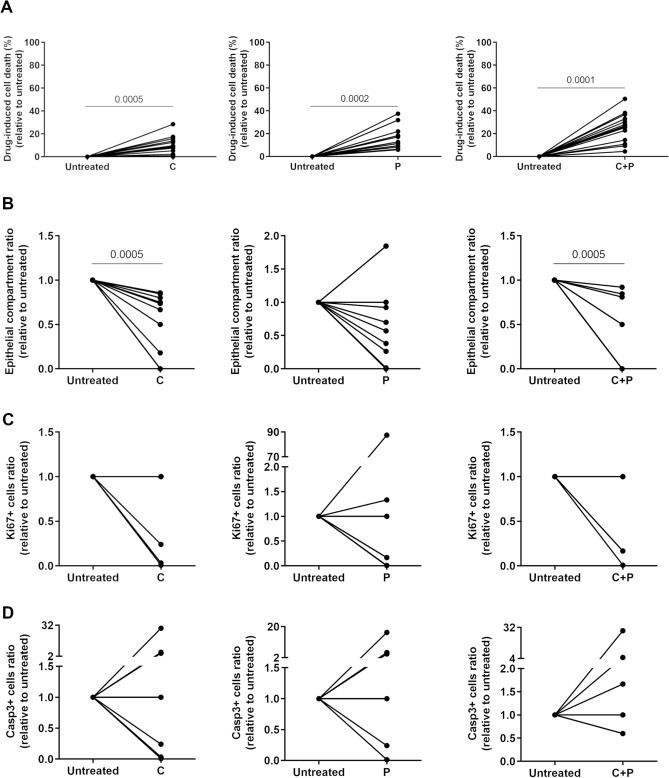
Drug-induced cell death variations are better captured by LDH than clinically-utilized endpoint readouts. Drug-induced cell death evaluation of OvC-PDE using (**A**) LDH assay (N = 13), (**B**) epithelial compartment (malignant tumor cells) ratio (N = 12), and (**C**) proliferation (N = 6–8) and (**D**) apoptosis (N = 6–9) levels. Wilcoxon signed-rank statistical test was applied to compare C + P with untreated condition for all readout methods (for statistically significant differences, *p*-values are indicated in the graphics, C: carboplatin; P: paclitaxel).

To validate the LDH assay as a measure of overall cell death (Fig. 6A) in PDE culture, we compared it with other readout methods currently used on primary patient samples in the clinic, namely H&E for tumor and stroma quantification and immunohistochemistry (IHC) analysis for evaluation of proliferation and apoptosis levels (Fig. 6, Supplementary Figs. 8 and 9, Supplementary Table 10). H&E staining revealed changes in histology, denoting differences in viable and necrotic areas and in the proportion of epithelial and stromal compartments (Supplementary Fig. 8), when comparing untreated and drug challenged explants. After two drug challenges, a significant reduction in the epithelial compartment was observed for SOC chemotherapy and carboplatin (*p* = 0.0005) and the same trend for paclitaxel (Fig. 6B). However, comparing the FC between the combination effect and HSA, the LDH assay and epithelial compartment ratio analysis do not agree in all cases (Supplementary Fig. 10). Moreover, there is a trend toward decreased levels of proliferation and increased levels of apoptosis after all drug challenges (Fig. 6C,D). Overall, response variability was best captured using the LDH assay compared with H&E and IHC readouts.

## Discussion

We have demonstrated the utility of the LDH assay to determine therapeutic efficacy in complex 3D cultures, namely xenospheres and PDE. Although PDE may recapitulate the clinical pathology, their applicability for drug discovery has been hindered by the limited validation and reproducibility of established readout methods to determine therapeutic efficacy in these culture systems^15,16^. Parameters beyond measures of cell content for analysis of therapeutic efficacy also need to be considered as greater complexity is introduced into preclinical tumor models.

As the time scale of therapeutic responses is highly variable, analytical methods that are non-invasive and allow for repeated observation of dynamic changes within the 3D cell culture are desirable^17^. Additionally, many efforts are being directed toward the development of in vitro cultures derived from primary patient samples. As patient material is highly limited, assays that can preserve samples, either through their compatibility with other readout methods or their ability to generate multiple data sets from the same sample, are needed. However, longitudinal determination of growth and viability in complex 3D cultures is challenging as many bioassays are developed for two-dimensional (2D) culture formats and designed as endpoint tests^17^. Some efforts toward assay validation in 3D monocultures are reported in the literature. Huber et al. analyzed cell viability, necrosis and proliferation in spheroids of a lung cancer cell line challenged with afatinib, cisplatin or vinorelbine. The authors adapted ATP, LDH and annexin V/propidium iodide (PI) staining, microscopic volume/diameter assessment and cell cycle analyses to 3D spheroid cultures. They highlighted the need for assay adaptation for each type of 3D culture, namely determination of total intra- and extra-cellular ATP and LDH content^18^.

The architectural complexity and heterogeneous content associated with 3D cultures also pose a challenge for cytotoxic assays to accurately report treatment outcomes^19^. Among common readout methods, the LDH assay, volume measurements, fluorescence and luminescence measurements of reporter protein-expressing cells have the benefit of longitudinal culture monitoring, enabling data collection without sacrificing the integrity of the sample. Unfortunately, the generation of luciferase-expressing cancer cells from the patient material will result in the loss of essential stromal and ECM components as well as the selection of a subpopulation of cancer cells, eliminating the heterogeneity that is crucial to modeling clinical responses^20–22^. Volume measurements are also limited in their inability to distinguish between live and dead cells and to account for the presence of necrotic areas within the spheroids, further restricting assessments of cell viability^23^.

We, therefore, endeavored to determine the utility of the LDH assay to analyze complex 3D cultures, particularly those derived from patient tumor tissues. LDH is a cytosolic enzyme released into the cell culture medium upon plasma membrane leakage, indicating cell toxicity^24^. LDH release has been used successfully to measure cell growth and viability in multicellular spheroids and bioreactor 3D culture systems^25–28^. However, standard protocols for accurate and reproducible quantification of therapeutic effects in complex ex vivo cultures, such as explant models, have yet to be described. We demonstrate the value of the LDH assay through measurements of cell growth and viability in spheroid cultures and cytotoxic agent efficacy in ex vivo cultures compared to other standard readout methods. Using spheroids, we showed that LDH assay measurements of cell growth and viability directly correlated with volume measurements, the CTG-3D assay, and luminescence measurements of luciferase-expressing cells. Spheroid cultures were used to generate sufficient sample number and uniformity. The confirmation of reliable and sensitive cell content measurements via the LDH assay in spheroid cultures, a basic 3D culture format, enabled us to expand its application to more complex models.

We used simulated data to assess the impact of culture dynamics on the determination of drug-induced cell death by LDH assay. Overall, cell growth and death dynamics and background LDH release might impact the accurate determination of cell death. Culture dynamics must be well characterized before the LDH assay can be effectively applied to complex culture systems. The culture format and objective of the drug-induced cell death evaluation will dictate the most appropriate method of data collection and analysis. If cultures are longitudinally monitored but have background levels of LDH or high rates of cell death, accurate IC50 values cannot be determined unless both control and treated samples are lysed (Eq. 5). Although drug-induced cell death based on CM samples and control group lysate (Eq. 3) may hinder the determination of precise IC50 during longitudinal culture monitoring, differences in therapeutic efficacy between treatment groups can still be determined, making stack ranking of compound efficacy a possibility.

We first applied the LDH assay to xenosphere cultures derived from NCI-H1650 and NCI-H1650.LMC xenografts treated with DTX. We demonstrated that the LDH assay is as effective as other commercially available cell viability readouts at determining therapeutic efficacy. The availability of cell line-derived xenografts for xenosphere generation is much less restricted than patient material, thus preserving the biological material is not as crucial^7,29–31^. When applied to the patient-derived culture system, the LDH assay captured a range of therapeutic responses, with the additional benefit of longitudinal culture monitoring. We further observed that variations in drug-induced cell death were best captured by the LDH assay compared to clinically utilized endpoint readouts (H&E and IHC). Although chemotherapeutic agents target cancer cells (epithelial compartment), widespread cell death in both epithelial and stromal compartments might occur, contributing to an overall decrease of tumor burden, which is not captured by the epithelial compartment ratio determined by H&E analysis. Moreover, H&E and IHC analyses are highly dependent on the cellular and tissue integrity at the end of the culture. In most OvC cases, after two drug cycles, few or no tumor cells were observed, which hinders the application of these readouts. Determinations of therapeutic efficacy might also be inaccurate using endpoint-based methods in long-term drug exposure assays since the remaining cells could represent a resistant population. In these situations, cell death and further detachment cannot be considered over the culture period.

## Conclusions

Altogether, the LDH assay is a suitable method for longitudinal monitoring and endpoint analysis of therapeutic efficacy in complex 3D in vitro tumor models, such as tumor spheroids, xenospheres and PDE. Moreover, it is not dependent upon the integrity of the sample at the experimental endpoint. However, culture dynamics limit the accuracy of drug-induced cell death determined by longitudinal measurements and must be considered when making conclusions of therapeutic efficacy. The development of standardized protocols to apply compatible readout methods, like the LDH assay, to complex 3D culture systems could greatly benefit preclinical and co-clinical studies. Reliable readout methods will enable the use of complex in vitro cultures, such as xenospheres, to test therapies targeting the tumor microenvironment or eliminate therapies that are likely to show no efficacy in vivo due to resistance mechanisms that can only be captured in a more complex culture system than the traditional monolayer^32–34^. The use of such culture systems in early drug discovery could save vast amounts of resources in the form of time and money. This study also demonstrated that the LDH assay applied to PDE cultures can recapitulate sample-specific response variability to SOC chemotherapeutic drugs and is suitable to study combinatorial effects of drug combinations. Even though further prospective studies should be performed to evaluate the prediction capability of the PDE cultures, this study foresees the broader application of LDH assay methodology as a therapeutic efficacy tool in future co-clinical assays for precision medicine and patient stratification.

## Methods

### 3D culture generation

#### Cell culture

Lung adenocarcinoma NCI-H1650 (ATCC, Manassas, VA) and NCI-H1650.LMC cells (NCI-H1650 cells transduced with luciferase and mCherry) were cultured in RPMI 1640 cell culture medium (Gibco, Gaithersburg, MD) supplemented with 10% fetal bovine serum (FBS, Fisher Scientific, Waltham, MA) and 1% penicillin/streptomycin (P/S, Fisher Scientific, Waltham, MA). To generate the NCI-H1650.LMC line, a fusion construct of luc2 (Promega, Madison, WI) and mCherry (Clontech, Mountain View, CA) was cloned into the Lenti-X lentiviral vector (Clontech). NCI-H1650 cells were transduced with lentiviral particles for 48 h and a pool of cells stably expressing the fusion construct were selected using 2 µg/ml puromycin for 2 weeks. All cultures were maintained in a 37 °C humidified incubator with 5% CO_2_. In all experiments, cells with less than 10 passages after thawing were used.

### Spheroids

#### Spheroid generation

A suspension of NCI-H1650 and NCI-H1650.LMC cells were seeded at 2500, 5000, 7500, 10,000, 12,500, and 15,000 cell/well of 96-well ultra-low attachment round bottom plates (Corning, Corning, NY), in 200 µl of culture medium per well. Plates were subsequently centrifuged at 335 × *g* for 5 min and cultured up to 7 days in a 37 °C humidified incubator with 5% CO_2_.

### Xenospheres

#### Xenosphere generation

NCI-H1650 and NCI-H1650.LMC cells suspended in culture medium were mixed (1:1) in Matrigel (Corning, Corning, NY). Five million cells in a total volume of 100 µl were injected subcutaneously in the right flank of ten female SCID-beige mice for each cell type (Charles River Laboratory, Wilmington, MA). Xenografts were collected when tumors reached a volume of 200–300 mm^3^. Excised tumors were placed in serum-free RPMI 1640 culture medium in a glass petri dish. Tumors were cut into pieces of approximately 500–600 µm using sterile scalpels and guided by the reticle of an Olympus SZX9 microscope (Olympus Life Science, Tokyo, Japan). Individual xenospheres were directly transferred into a single well of an ultra-low attachment round bottom 96-well plate and cultured in 200 µl of RPMI 1640 culture medium supplemented with 10% FBS and 1% P/S. Xenospheres were cultured up to 7 days in a 37 °C humidified incubator with 5% CO_2_.

#### Animal husbandry

Seven-week-old mice were obtained from Charles River (Wilmington, MA). Ten mice were housed per cage. The body weight upon arrival was 18–20 g. Food and water were available ad libitum. Mice were acclimated to the animal facilities for a period of at least 1 week prior to the commencement of experiments. Animals were tested in the light phase of a 12-h light: 12-h dark schedule. Mice were individually tagged and assigned to numbered cages. At the study endpoint, animals were humanely euthanized via isoflurane inhalation followed by exsanguination. All experiments were conducted in compliance with AbbVie’s Institutional Animal Care and Use Committee and the National Institutes of Health Guide for Care and Use of Laboratory Animals guidelines in a facility accredited by the Association for the Assessment and Accreditation of Laboratory Animal Care (AAALAC). All methods are reported in accordance with ARRIVE guidelines.

### PDE

#### Study sample

Consent to fresh surgically removed tumors from 16 patients with OvC who underwent surgery at Instituto Português de Oncologia de Lisboa, Francisco Gentil (IPOLFG) was obtained from 2018 to 2020. Tumor specimens were transported in DMEM culture medium from the surgery room to the laboratory. Samples were named chronologically from OvC1 to OvC16 (Supplementary Table 7).

#### Sample collection and processing

Fresh (up to 4 h) surgically removed tumor specimens were weighed and mechanically dissociated into fragments of approximately 1 mm^2^ as recently described by our team^13^.

### Cell viability assays in spheroid cultures

Cell viability assays were performed on days 0 (day after seeding) and 7 of spheroid culture.

#### Volume measurements

Brightfield images of spheroids were collected on a Nikon Eclipse Ti at 4x. Area of the imaged spheroid section was determined by ImageJ analysis. Volume was calculated as:

1 $${\text{Volume}} = \frac{4}{3}\sqrt {\frac{{{\text{Area}}^{{ 3}} }}{\uppi }}$$

#### LDH assay

LDH activity was measured using the Pierce LDH Cytotoxicity Assay Kit following the manufacturer protocol (ThermoFisher Scientific, Waltham, MA). Briefly, 50 µl of conditioned medium (CM) was collected from each sample. The cells were lysed using 10 µl of the lysate buffer provided in the assay kit, for 45 min at 37 °C and 50 µl of lysed cells were collected from each sample. The LDH assay was run on the CM and lysate samples. Absorbance was measured at 490 and 680 nm on a SpectraMax iD5 (Molecular Devices, San Jose, CA).

#### CTG-3D assay

ATP content in spheroid cultures was assessed using a CTG-3D Cell Viability Assay (Promega, Madison, WI) following the manufacturer protocol. Luminescence was measured on a SpectraMax iD5.

#### Luminescence measurements

Luminesce measurements were taken from NCI-H1650.LMC spheroid cultures. A 15 mg/ml solution of D-luciferin potassium salt [PerkinElmer, Waltham, MA] in sterile phosphate-buffered saline (PBS, Fisher Scientific, Waltham, MA) was added to each sample medium to generate a final luciferin concentration of 1.5 mg/ml. Samples were protected from light and placed in a 37 °C, 5% CO_2_ incubator for 1 h. Luminescence of each sample was measured on a SpectraMax iD5.

#### Assessment of spheroid culture growth

We utilized a normalized growth rate equation adapted from Hafner et al.^35^: to determine growth rate inhibition following drug treatments, to compare cell growth in the spheroid cultures as determined by each readout method

2 $${\text{GR}}\left( {{\text{ro}}} \right) = 2^{{\log_{2} \left( {\frac{{{\text{x}}_{{{\text{ro}}}} }}{{{\text{x}}_{{\text{0, ro}}} { }}}} \right)/\log_{2} \left( {\frac{{{\text{x}}_{{{\text{ctrl}}}} }}{{{\text{x}}_{{\text{0, ctrl}}} }}} \right)}} - 1$$

Instead of cell number as previously described, x_0, ro_ and x_ro_ represent the cell content at the beginning and end of the experiment, respectively, as determined by the readout method of interest. X_0, ctrl_ and x_ctrl_ represent the cell content at the beginning and end of the experiment, respectively, as determined by a control readout. Absorbance, luminescence, or volume measurements at assay timepoints were used to measure cell content. Volume measurements were used as the control readout method.

### LDH assay simulation

LDH data was simulated to determine how growth rate and background LDH release in untreated samples may impact the evaluation of therapeutic efficacy in 3D cultures. Values for LDH content in the cell lysate and CM were generated to reflect cases of no growth or cell tripling over 7 days in culture with 0% or 10% background LDH release following treatments that induce 0–80% cell death over the culture period (7 days) or daily. Drug-induced cell death values were simulated by three equations:

3 $$\%{\text{ drug}}{ - }{\text{induced}}\;{\text{death}}\;{\text{A}} = \left[ {\frac{{{\text{CM}}\;{\text{LDH}}\;{\text{treated}} - {\text{CM}}\;{\text{LDH}}\;{\text{control}}}}{{{\text{Total}}\;{\text{LDH}}\;{\text{control }}}}} \right] \times 100$$

4 $$\%{\text{ drug}}{ - }{\text{induced}}\,{\text{death}}\;{\text{B}} = \left[ {\frac{{{\text{CM}}\;{\text{LDH}}\;{\text{treated}}}}{{{\text{Total}}\;{\text{LDH}}\;{\text{treated}}}} - { }\frac{{{\text{ CM}}\;{\text{LDH}}\;{\text{control}}}}{{{\text{Total}}\;{\text{LDH}}\;{\text{control }}}}} \right] \times 100$$

5 $$\%{\text{ drug}}{ - }{\text{induced}}\;{\text{death}}\;{\text{C}} = \left[ {1 - \frac{{{\text{Lysate}}\;{\text{LDH}}\;{\text{treated}}}}{{{\text{Lysate}}\;{\text{LDH}}\;{\text{control}}}}} \right] \times 100$$

where total LDH is the sum of the CM and lysate LDH values.

According to the manufacturer protocol, only CM and lysate from the control group should be considered (Eq. 3). To address cell growth and LDH background release, lysates from control and treated groups were considered together with the CM (Eq. 4) or only lysate content was considered (Eq. 5).

### SOC therapeutic efficacy in ex vivo cultures

#### Assessment of DTX efficacy in xenosphere cultures

DTX (Sigma-Aldrich, St. Louis, MO) treatments of control, 0.1, 5, 25, 125, 625, and 3125 nM were added to xenospheres on the day of xenosphere generation (day 0). Samples were treated for 7 days. DTX efficacy was assessed in NCI-H1650 xenospheres via volume measurements, LDH assay, and CTG 3D assay. DTX efficacy was assessed in NCI-H1650.LMC xenosphere cultures via volume and luminescence measurements. Control samples on day 0 were also assessed via the cell line’s respective readout methods. All readout methods were carried out as described previously for the spheroid cultures. The half-maximal inhibitory concentration (IC50) values were determined by four-parameter variable slope logistic regression analysis of the concentration-response data.

#### Drug challenge in PDE cultures

Drug challenges were conducted in PDE cultures of 10 PDE/well in 12 well plates at a concentration of 5 PDE/ml under orbital agitation at 100 rpm. OvC-PDE cultures from different OvC subtypes were challenged weekly with SOC chemotherapy, namely carboplatin (25 mg/ml) or paclitaxel (10 mg/ml) as single agents or in combination^13,36,37^. Briefly, after 7 days of culture, the medium of all wells was exchanged and OvC-PDE were challenged with single agents or a pre-mixed drug combination for 1 week. At the end of the first drug cycle (day 14 of culture), a complete exchange of medium followed by a second drug challenge for one additional week was performed (until day 21 of culture). Cell death was assessed longitudinally (day 7, 14, and 21 of culture) by LDH assay. Additionally, morphology and quantification of epithelial (malignant tumor cells) and stromal compartments were assessed by histopathological analysis and proliferation and apoptosis levels by immunohistochemistry.

#### Cell death evaluation by LDH assay in PDE cultures

CM was collected from each sample on days 7, 14, and 21 of culture. At each timepoint, PDE from at least one control well were lysed with 10% triton X-100 for 24 h. Culture media from a well without cells was collected to determine the LDH levels in the sera, which was discounted from all CM conditions. Following CM collection, samples were centrifuged at 1000 × *g* for 5 min, at 4 °C, and stored at 4 °C until further analysis (up to 3 days). For the LDH assay, 50 µl of each sample (previously diluted in PBS to ensure an absorbance measurement within the linear range) was transferred to a 96-well flat-bottom plate in technical duplicates. The LDH assay was performed as described above. To calculate % drug-induced cell death, the following equation was used:

6 $$\%{\text{ drug}}{ - }{\text{induced}}\;{\text{cell}}\;{\text{death}} = \sum_{{{\text{cycle}}}} \left[ {\frac{{{\text{CM}}\;{\text{LDH}}\;{\text{treated}}}}{{{\text{Total}}\;{\text{LDH}}\;{\text{control}}}} \times \%_{{{\text{at}}\;{\text{start}}\;{\text{of}}\;{\text{cycle,}}\;{\text{treated}}}}^{{{\text{live}}\;{\text{cells}}\;{\text{remaining }}}} - \frac{{{\text{CM}}\;{\text{LDH}}\;{\text{control}}}}{{{\text{Total}}\;{\text{LDH}}\;{\text{control}}}} \times \%_{{{\text{at}}\;{\text{start}}\;{\text{of}}\;{\text{cycle,}}\;{\text{control}}}}^{{{\text{live}}\;{\text{cells}}\;{\text{remaining }}}} } \right]{ }$$

#### Histopathological analysis

The morphology of parent patient tumor tissue was compared with the corresponding OvC-PDE at day 0 of culture (after processing) and at the end of the culture (day 21). Samples were processed for H&E staining and quantified as previously described^13^.

#### IHC analysis

Proliferation and apoptosis levels of original OvC tumors were compared with corresponding OvC-PDE on day 0 of culture (after processing) and at the end of the culture (day 21). OvC-PDE from untreated control and drug-treated conditions were also assessed at the end of the drug challenge (day 21 of culture). Samples were processed and analyzed as previously described^13^. The predominant intensity of staining was recorded on a scale of 0–100%, divided into six bins: < 1, 1–5, 5–25, 25–50, 50–75, > 75%. Median bin values, assessed at the end of drug challenge (day 21 of culture), were considered for further comparison of untreated controls and drug-treated conditions.

### Statistical analysis

Statistical analysis was performed using one-way ANOVA, Tukey’s test for multiple comparisons, Sidak’s test for multiple comparisons, Pearson correlation analysis, and Wilcoxon signed-rank statistical test. GraphPad Prism version 7.05 software was used for all analysis. Results were considered statistically significant for *p* < 0.05 and were expressed as mean ± standard deviation (SD).

### Ethics approval and consent to participate

The animal studies and all procedures involved were approved by the AbbVie’s Institutional Animal Care and Use Committee, in compliance with the rules of the National Institutes of Health Guide for Care and Use of Laboratory Animals guidelines. All procedures performed in studies involving human participants were in accordance with the ethical standards of the institutional and/or national research committee. Informed consent was obtained from all individual participants prior to the enrollment in the study. Anonymized patient tumor samples were obtained from the IPOLFG after institutional review board approval. The project was approved by the Research Council of IPOLFG and by the Ethics Committee for Health of IPOLFG (UIC-1088 and UIC-1211).

### Consent for publication

All authors have given consent for publication.

## Supplementary Information

Supplementary Information.

## Data Availability

The datasets used and/or analyzed during the current study are available from the corresponding author on reasonable request.
