# Supplementary material for: Application of LDH assay for therapeutic efficacy evaluation of ex vivo tumor models
Source: Sci Rep. 2021 Sep 17;11:18571. doi: 10.1038/s41598-021-97894-0 (PMC8448883; doi:10.1038/s41598-021-97894-0)
Supplement: Supplementary file 1 — Supplementary Information. [file 41598_2021_97894_MOESM1_ESM.docx]

**SUPPLEMENTARY INFORMATION**

**Application of LDH Assay for Therapeutic Efficacy Evaluation of Ex Vivo Tumor Models**

Megan C. Cox^1,^ *, Rita Mendes^2,3,^ *, Fernanda Silva^4^, Teresa F. Mendes^2,3^, Adelyn Zelaya-Lazo^1^, Kathleen Halwachs^1^, Julie J. Purkal^1^, Inês A. Isidro^2,3^, Ana Félix^4,5^, Erwin R. Boghaert^1^, Catarina Brito^2,3,6 +^

^1^AbbVie

1 North Waukegan Road, North Chicago, IL 60064-6098, United States of America

^2^IBET, Instituto de Biologia Experimental e Tecnológica

Apartado 12, 2780-901 Oeiras, Portugal

^3^Instituto de Tecnologia Química e Biológica António Xavier, Universidade Nova de Lisboa

Avenida da República, 2780-157 Oeiras, Portugal

^4^CEDOC-FCM-NOVA, Centro de Estudos de Doenças Crónicas da Faculdade de Ciências Médicas, Universidade Nova de Lisboa

R. Câmara Pestana 6, 1150-078 Lisboa, Portugal

^5^IPOLFG, Instituto Português de Oncologia de Lisboa Francisco Gentil

R. Prof. Lima Basto, 1099-023 Lisboa, Portugal

^6^The Discoveries Centre for Regenerative and Precision Medicine, Lisbon Campus

Av. da República, 2780-157 Oeiras, Portugal

*Megan C. Cox and Rita Mendes contributed equally to this work.

^+^Corresponding author: anabrito@ibet.pt

1. **Methods**

***Spheroids***

*Spheroid Dissociation*

Spheroids were dissociated by first removing medium from the sample wells and rinsing the samples in PBS. The PBS was removed and TrypLE [Gibco, Thermo Fisher, Waltham, MA] was added to the spheroids. Spheroids were incubated at 37°C for 5 minutes. Spheroid samples were pipetted vigorously and sufficient culture medium to neutralize the TrypLE was added to each dissociated sample.

*MTS Assay*

Cell content in spheroid cultures was assessed on day 0 (day after seeding) and day 7 using an MTS Cell Proliferation Colorimetric Assay Kit following the manufacturer protocol (BioVision Inc, Milpitas, CA). Samples were incubated in MTS reagent for 4 hours prior to absorbance readings on a SpectraMax iD5 (Molecular Devices, San Jose, CA). Medium control samples (medium from wells without spheroids) were collected at each timepoint.

*PrestoBlue Assay*

Cell content in spheroid cultures was assessed on day 0 (day after seeding) and day 7 using a PrestoBlue Cell Viability Reagent Kit following the manufacturer’s protocol (Invitrogen, Carlsbad, CA). Samples were incubated in PrestoBlue reagent for 4 hours prior to fluorescence readings on a SpectraMax iD5. Medium control samples (medium from wells without spheroids) were collected at each timepoint.

***LDH Assay Simulation***

LDH data was simulated to determine how growth rate and background LDH release in untreated samples may impact the evaluation of therapeutic efficacy in 3D cultures. Values for LDH content in the lysate and media were generated to reflect cases of no growth or cell tripling and no background LDH release or 10% background LDH release for treatments that induce 0-80% cell death daily. Cell death values were determined by three methods as described in the manuscript.

***Xenospheres***

*Histology*

Xenospheres were collected on day 0, 3, and 7 of culture. A sample of the parent xenograft was also collected on day 0. Samples were fixed in 10% neutral buffered formalin. Prior to paraffin embedding, xenospheres were stained with 1% Alcian Blue (in 3% glacial acetic acid in water, pH 2.5) overnight at 4°C then washed in PBS to remove excess stain. Samples were cut into 4 µm serial sections and stained with H&E.

***PDE***

*Downscaling of OvC-PDE Cultures*

We have recently described the methodology for OvC-PDE cultures in 125 mL shake flasks, at 5 explant/mL, in 20 mL of culture medium (100 PDE)^1^. To best utilize the limited fresh patient samples, we have downscaled the culture. PDE were cultured in 125 ml shake flasks (50 PDE) or in 12 well-plates (10 and 5 PDE) at a concentration of 5 PDE/ml, under orbital agitation at 100 rpm. OvC-PDE were cultured in DMEM supplemented with 10% FBS and 1% P/s at 37°C and 5% CO_2_. OvC-PDE were cultured for 21 days and a complete medium change occurred every 7 days. OvC-PDE were collected on day 0 (surgery day, after tissue processing) and day 21 of culture for analysis of morphology and proliferation/cell death, namely apoptosis, levels by histopathology and immunohistochemistry, respectively. In addition, conditioned media was collected on day 7, 14 and 21 for longitudinal evaluation of cell death by the LDH assay.

**References:**

1. Abreu, S. et al. Patient-derived ovarian cancer explants: preserved viability and histopathological features in long-term agitation-based cultures. Sci. Rep. **10**, 1–13 (2020).

1. **Figures and Tables**

**
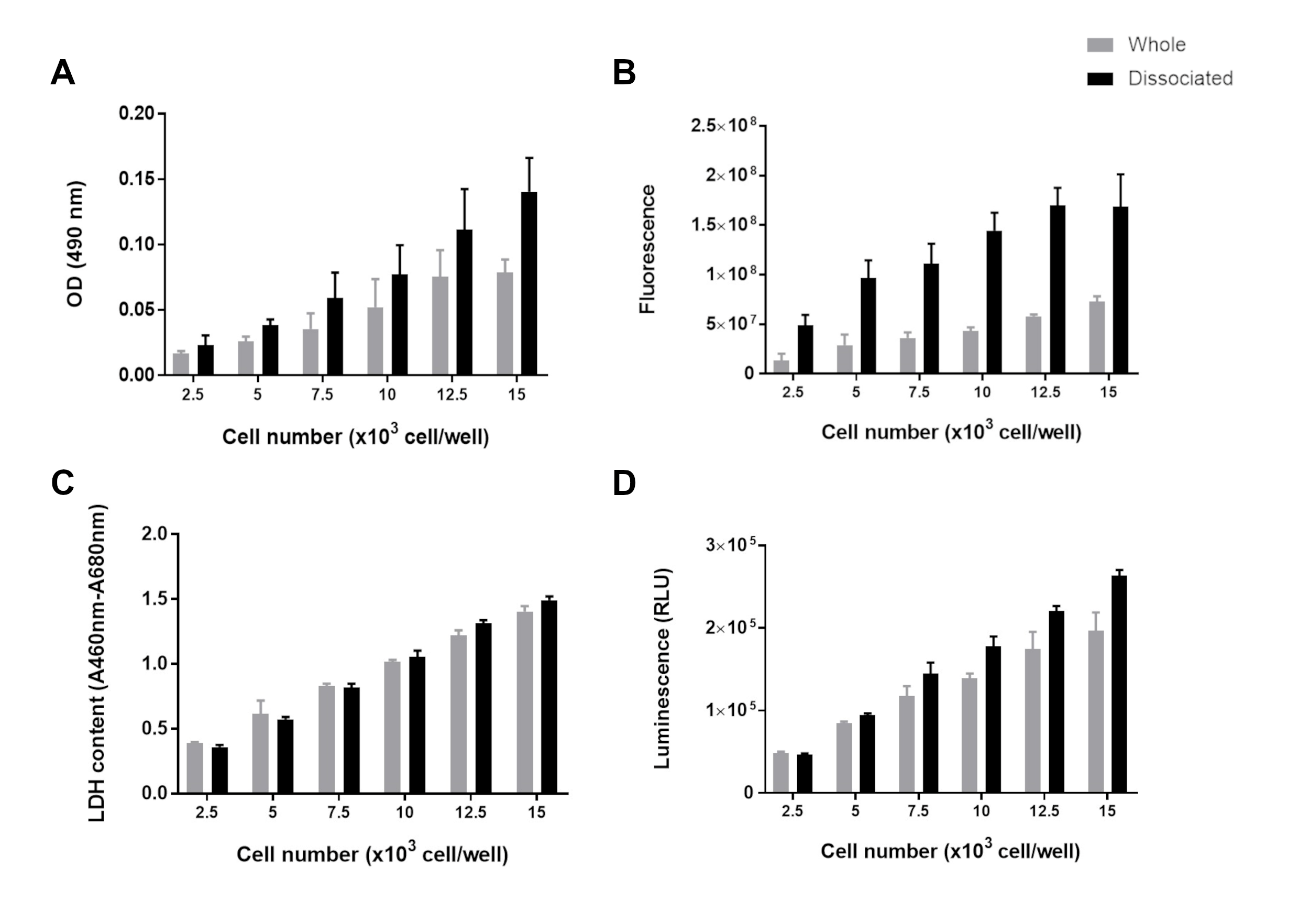
**

**Supplementary Figure 1. Reagent penetration variably impacts assay sensitivity.** (A) MTS, (B) PrestoBlue, (C) LDH content, and (D) CTG-3D measurements were collected from whole or dissociated NCI-H1650 spheroids composed of 2,500, 5,000, 7,500, 10,000, and 12,500 cells on day 0 of culture. Statistics for data presented in A-D is summarized in Supplementary Table 4-6. Data is presented as mean ± SD of N=5.


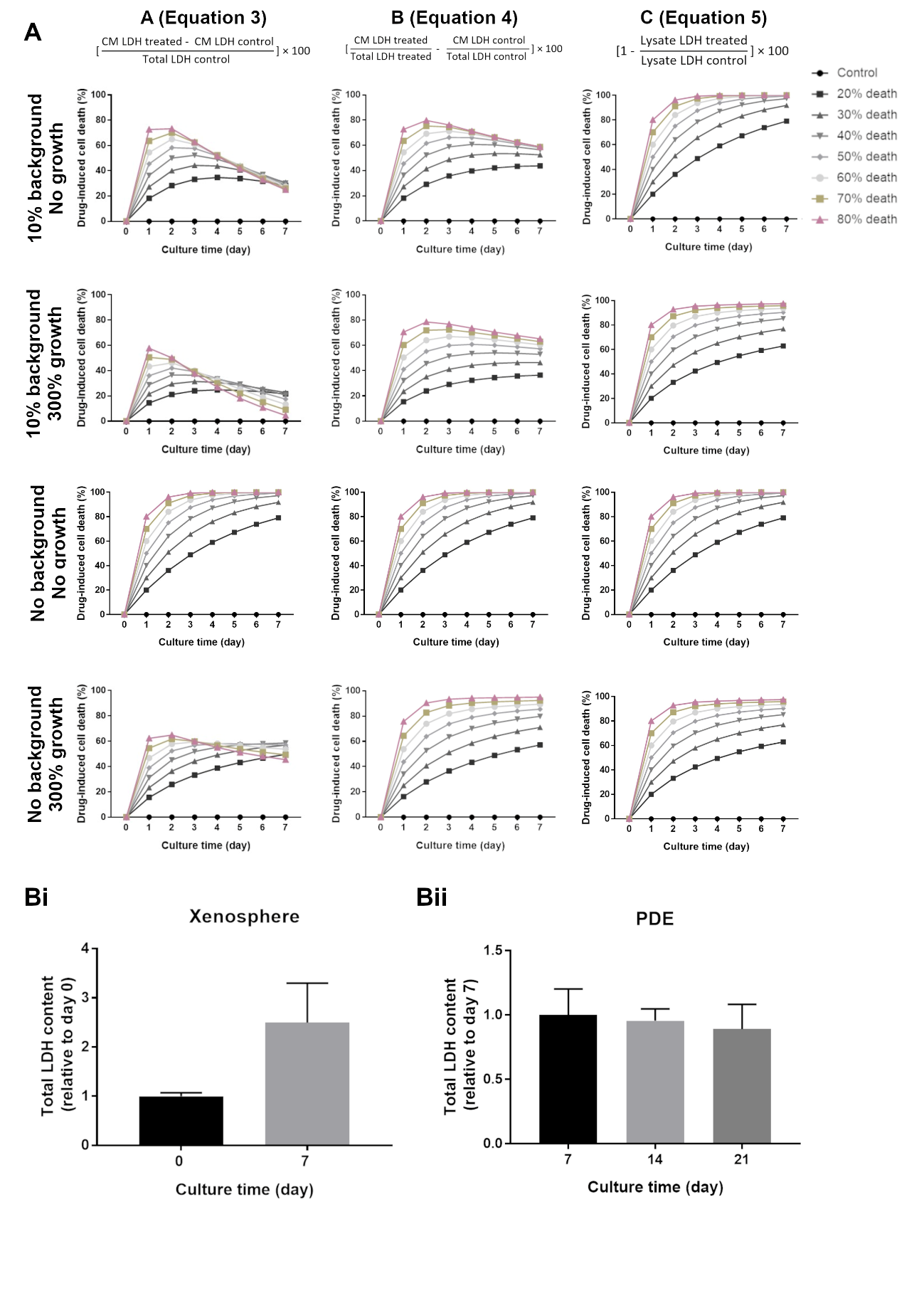


**Supplementary Figure 2. Culture growth dynamics impact how LDH data should be analyzed to evaluate therapeutic efficacy.** (A) Simulations represent cases in which a therapy that induces 0%-80% cell death daily has been applied to cultures that have 10% or no background LDH and do not grow or cells triple over the culture period. (B) Cell growth in (i) xenospheres and (ii) OvC-PDE cultures as determined by LDH content (data is presented as mean ± SD of N=5).


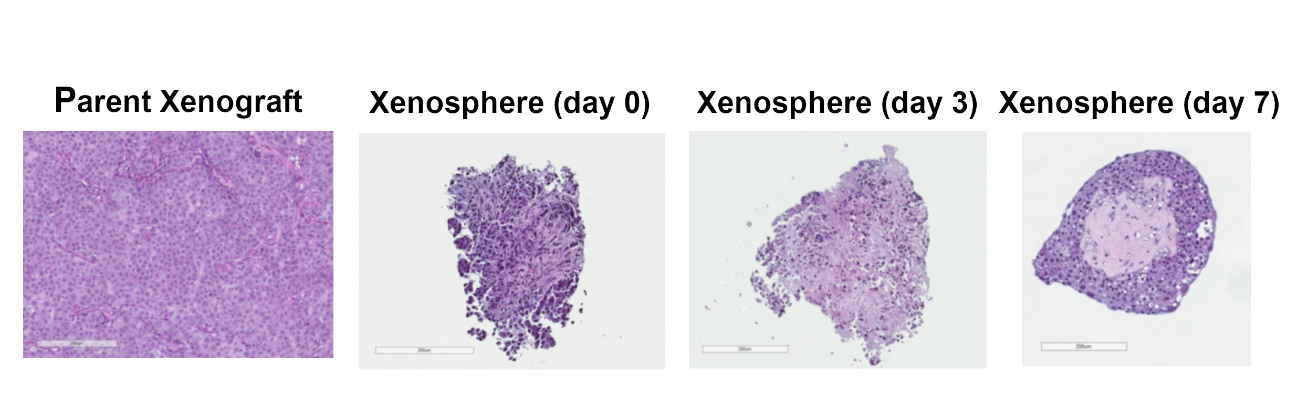


**Supplementary Figure 3. Morphology of parent xenograft and xenospheres over culture.** Images of H&E-stained samples of the parent xenograft and xenospheres on day 0, 3, and 7 of culture.


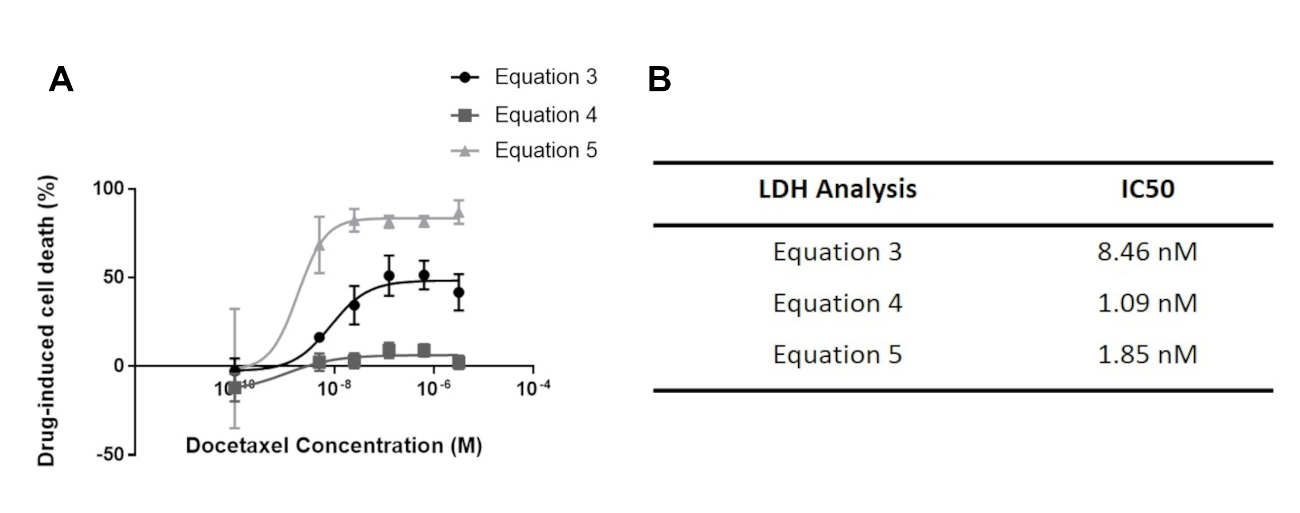


**Supplementary Figure 4. Equation 5 for LDH assay analysis is best suited for xenosphere cultures** (A) DTX efficacy in NCI-H1650 xenosphere cultures as determined by the three LDH assay analysis methods (data is represented as mean ± SD of N=5), (B) IC50 values of DTX in the xenosphere cultures as determined by each analysis method.


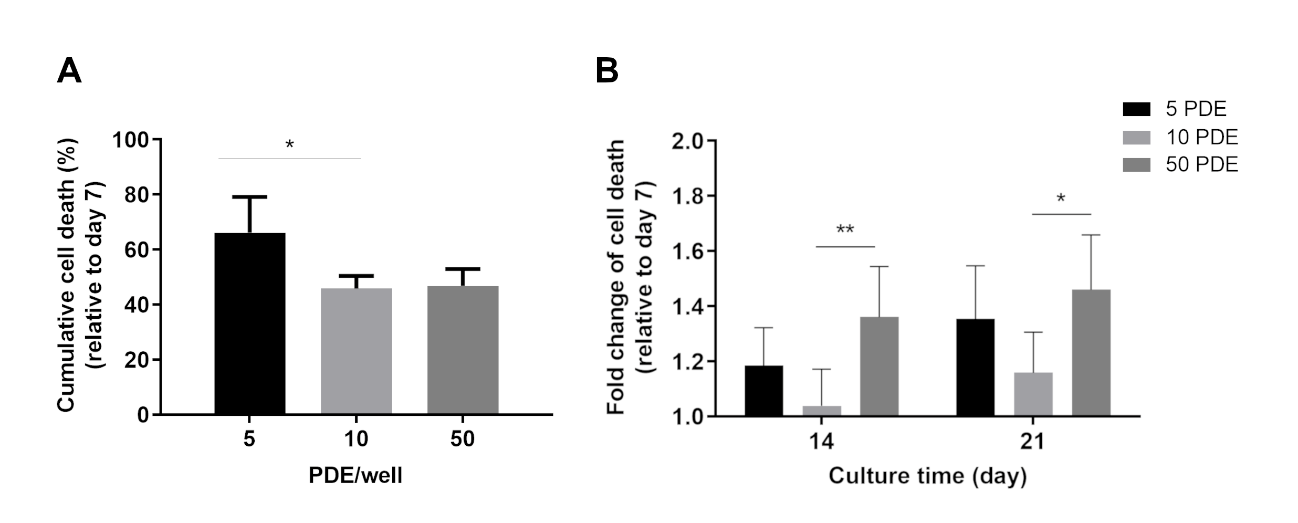
**Supplementary Figure 5:** **OvC-PDE viability evaluated by LDH leakage assay.** (A) At day 7 (before drug challenge) and (B) at the end of each time point of the drug challenge (day 14 and 21 of culture). Data is presented as mean ± SD of N ≥ 3. ANOVA statistical test was applied to compare LHD activity along culture period. * (p<0.05); ** (p<0.01).


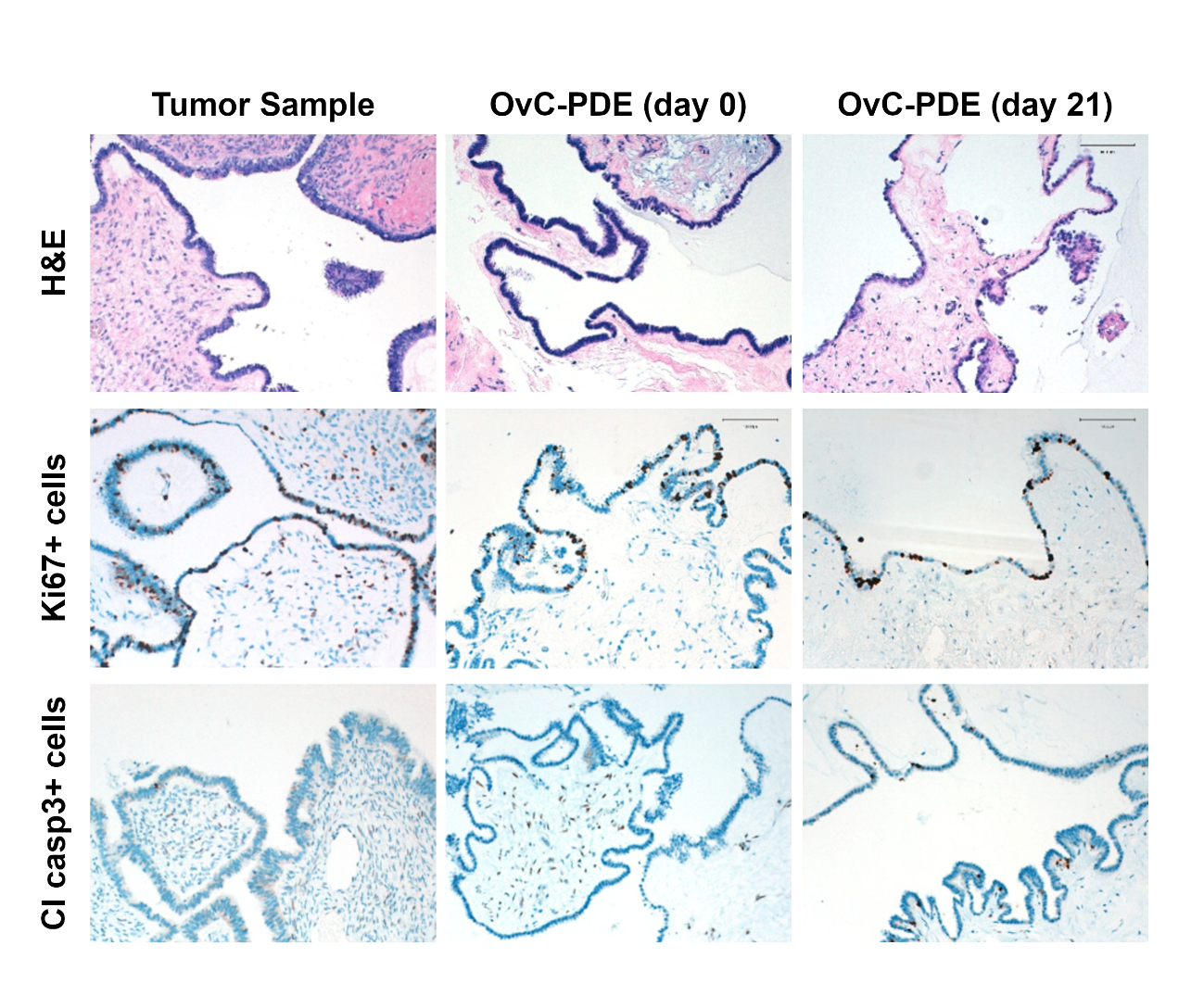
**Supplementary Figure 6: Architecture and features of the original clinical specimens were maintained throughout PDE culture (10 PDE/well).** Representative images (OvC2) of H&E staining and immunohistochemistry analysis (markers: Ki67 for proliferation and cleaved caspase 3 for apoptosis) of cross-sections of OvC-PDE after tissue processing (Day 0) and by day 21 of culture compared to original tumor samples. Scale bars represent 100 µm.

**
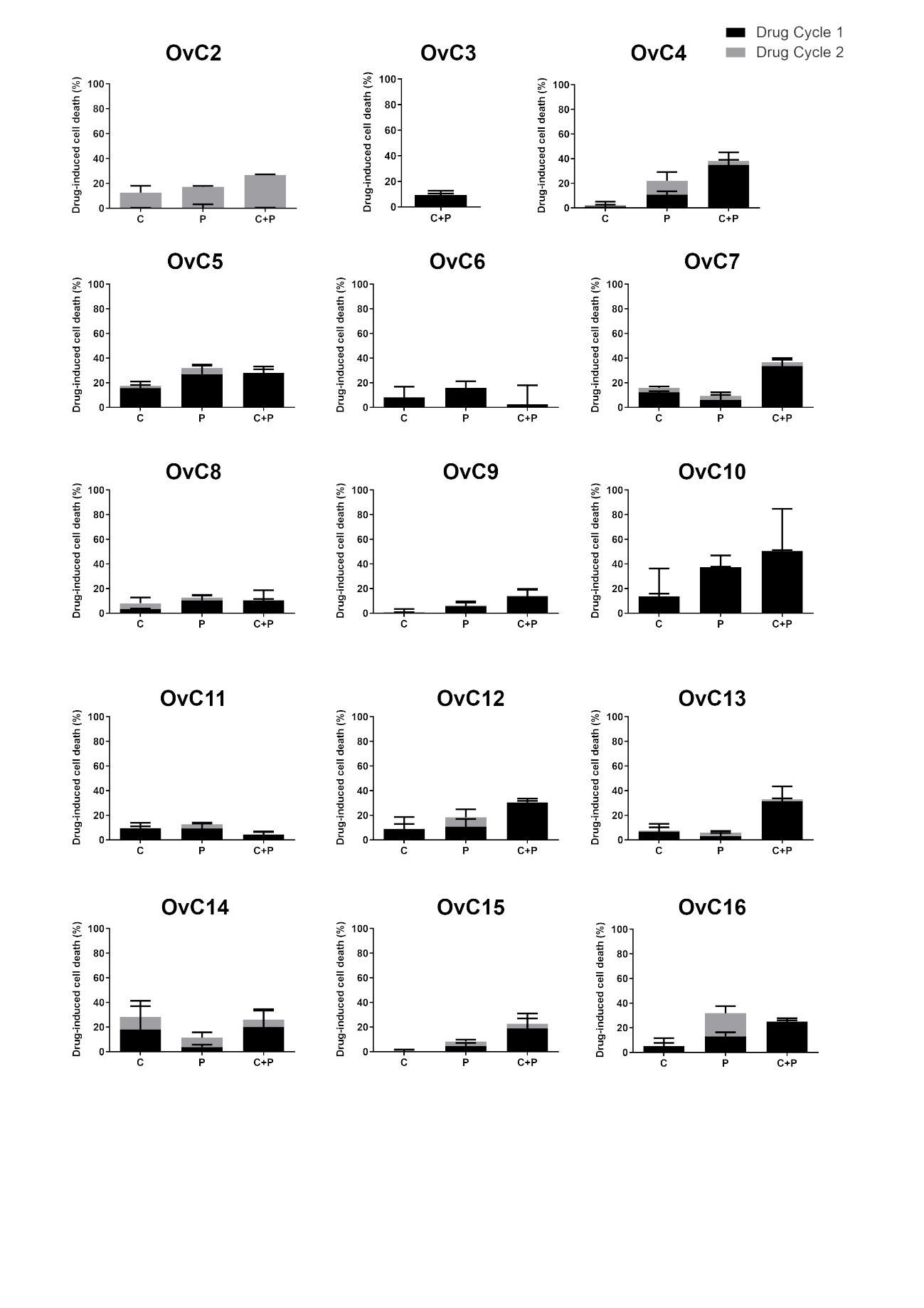
**

**Supplementary Figure 7:** **Drug-induced cell death of OvC-PDE along two drug cycles, evaluated by the LDH assay.** Data is presented as mean ± SD of three technical replicates per OvC-PDE case. (N=15, C: carboplatin, P: paclitaxel). OvC1 was only used for culture optimization purposes; for OvC3, tumor sample was small and therefore single agents could not be assessed; for OvC6, the tumor sample was composed exclusively of stromal compartment.


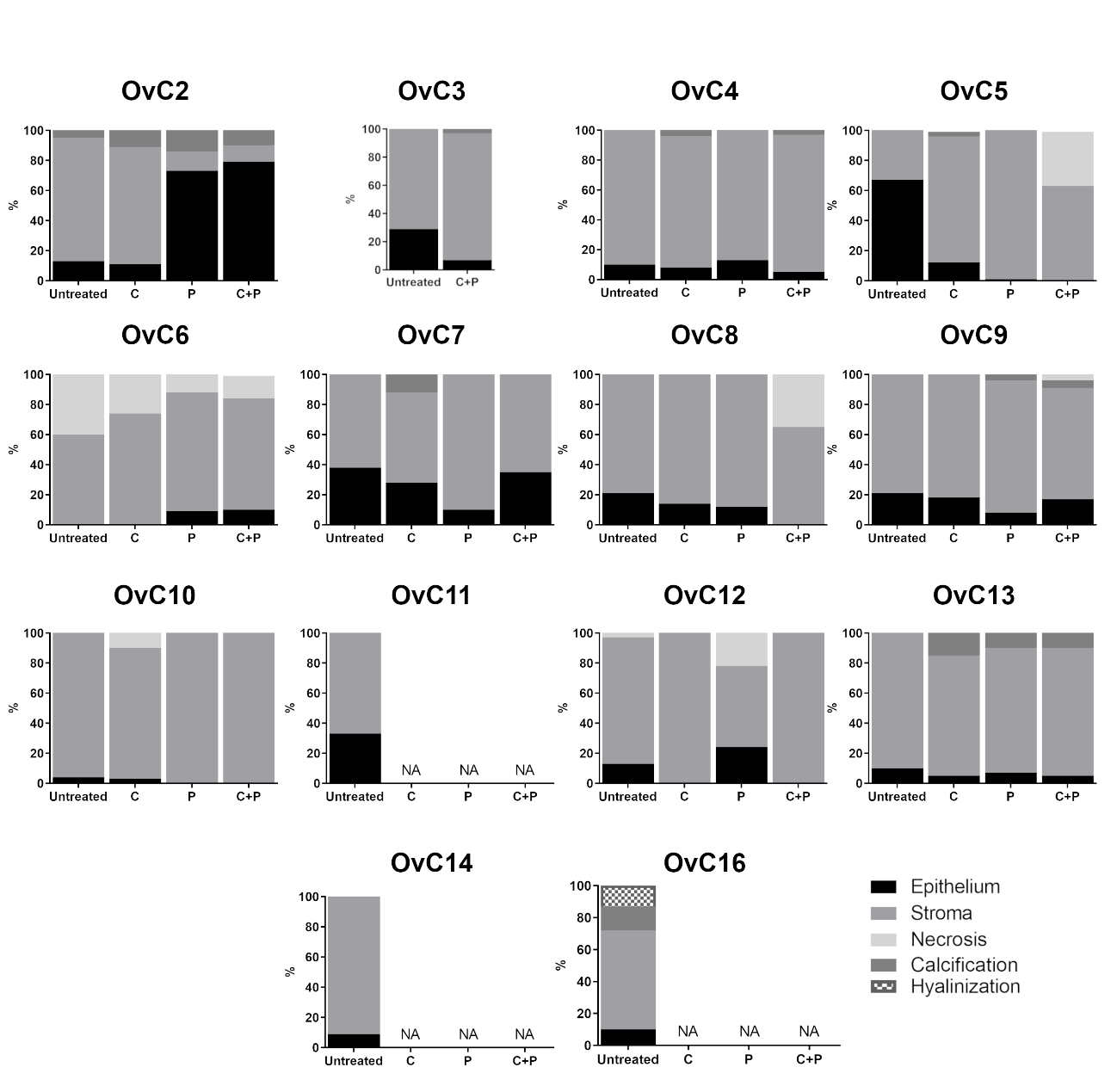


**Supplementary Figure 8:** **Epithelial and stromal proportions upon drug challenges** **by OvC-PDE case.** Percentage of epithelium (malignant tumor cells, black), stroma (grey), calcification (dark grey), necrosis (light grey) and hyalinization (dotted grey) at the end of the culture (day 21) (N=12; C: carboplatin; P: paclitaxel; NA: not available). Tissue integrity of OvC11, OvC14 and OvC16 was highly affected and therefore, 0% of epithelial compartment was considered for ratio calculations in these cases. OvC15 was discarded since scarce tumor cells were found in the untreated condition.


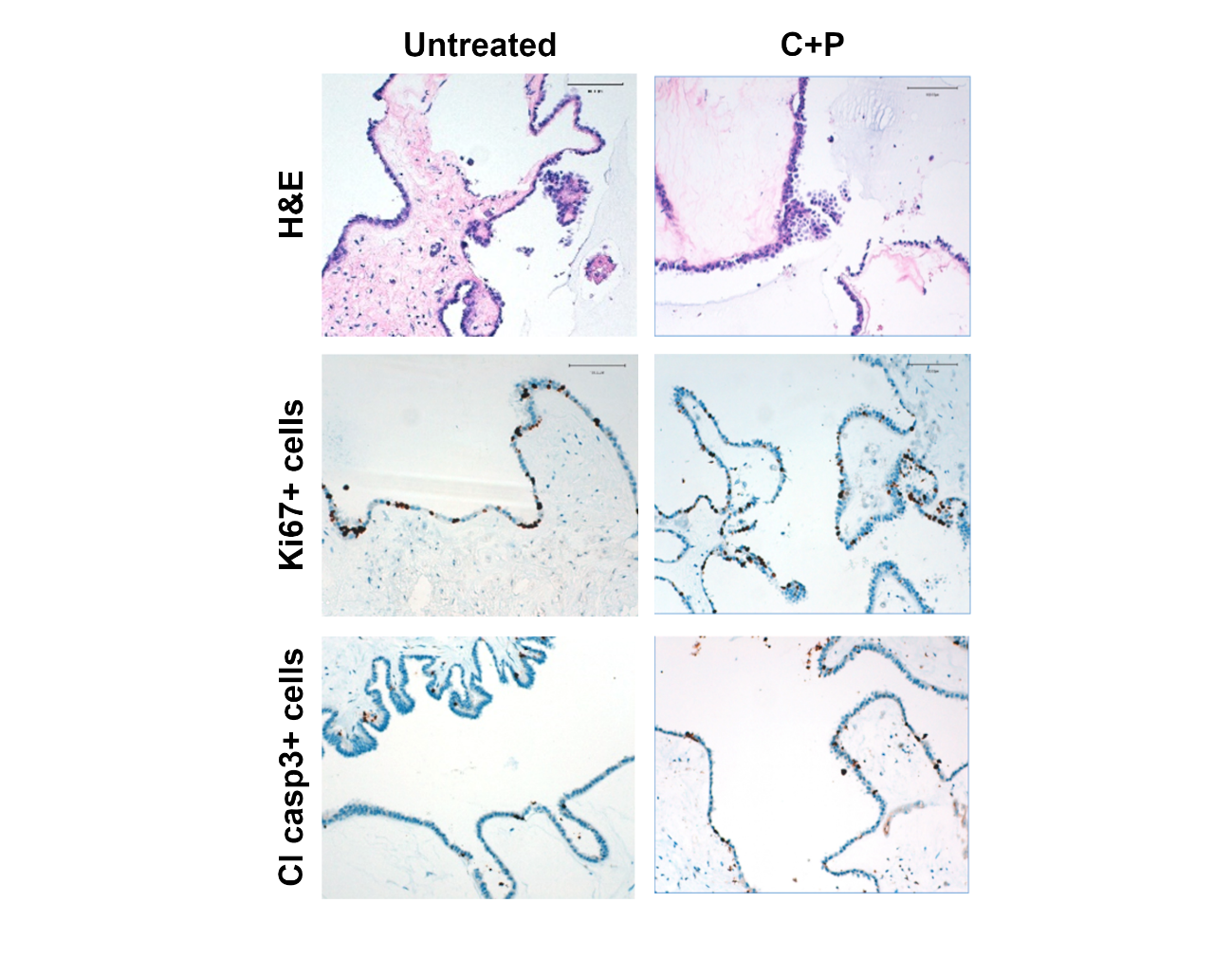
**Supplementary Figure 9:** **Drug-induced cell death evaluation of OvC2 on proliferation and apoptosis levels.** Representative images of Hematoxylin & Eosin (H&E) staining and immunohistochemistry analysis (markers: ki67 for proliferation and cleaved caspase 3 for apoptosis) of cross-sections of OvC-PDE at day 21 of culture. Scale bars represent 100 µm.


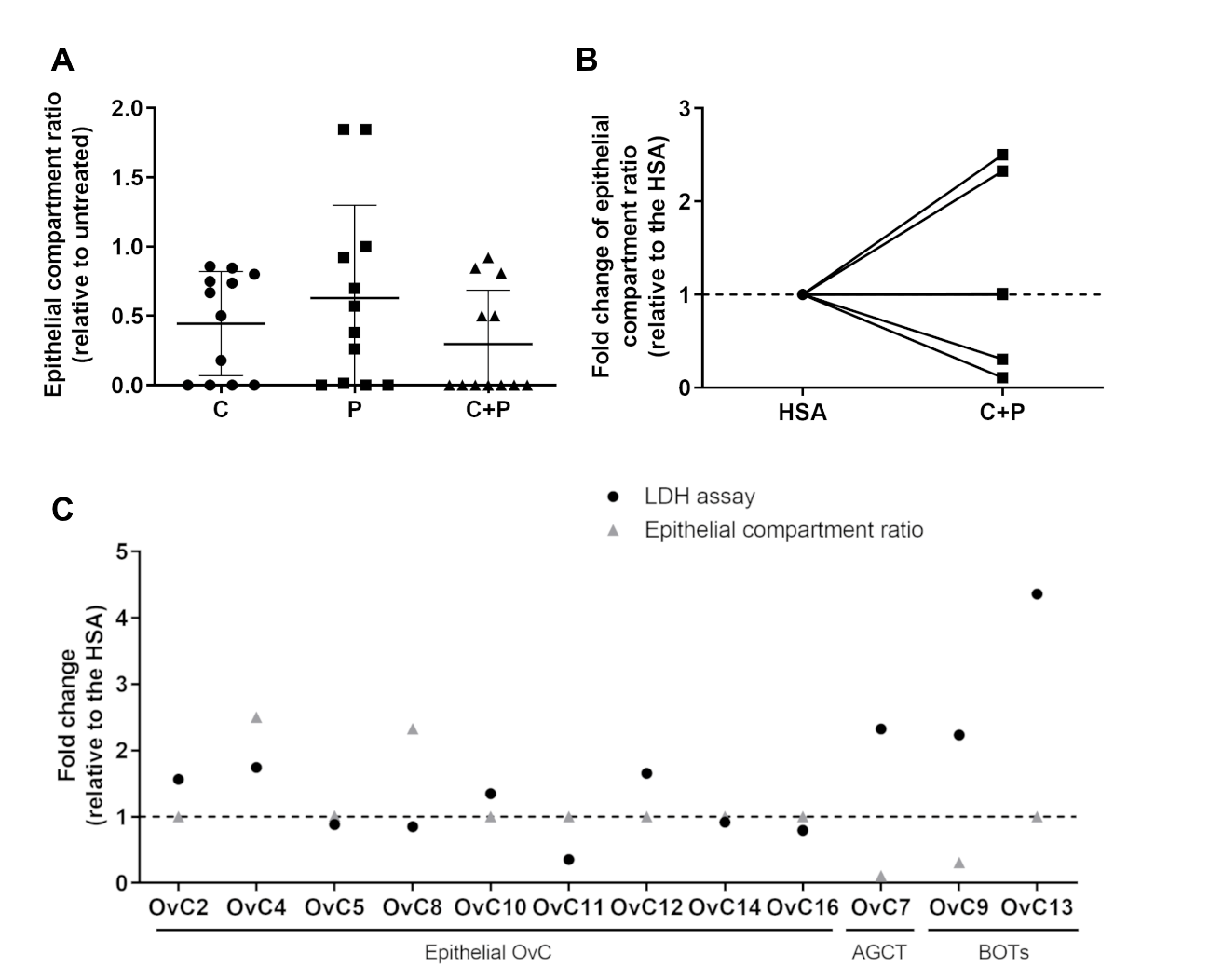


**Supplementary Figure 10**: **SOC chemotherapy and single drug efficacy in PDE cultures evaluated by epithelial compartment ratio.** (A) Epithelial compartment (malignant tumor cells) ratio over untreated at day 21. Data is presented as mean ± SD of N=12. (B) Fold change of Epithelial compartment ratio observed for SOC chemotherapy (C+P) over the highest single agent (Carboplatin for OvC2, OvC4, OvC11, OvC12 and OvC13 and Paclitaxel for the other OvC cases) (N=12). (C) Comparison between the fold change of SOC chemotherapy (C+P) over the highest single agent determined by LDH assay and epithelial compartment ratio. (N=12) (C: carboplatin, P: paclitaxel, AGTC: Adult Granulosa Cell Tumor; BOTs: Borderline Ovarian Tumors)

**Supplementary Table 1:** Summary of Tukey’s multiple comparisons test on LDH lysate, CTG-3D, volume, and luminescence day 0 measurements of NCI-H1650 and NCI-H1650.LMC spheroids.

| **Day 0** | **Significant (p-value)** | | | |
| --- | --- | --- | --- | --- |
|  | **LDH Lysate** | **CTG-3D** | **Volume** | **Luminescence** |
| **2500 vs 5000** | No (0.1134) | No (0.2916) | No (0.1497) | No (0.0796) |
| **2500 vs 7500** | Yes (<0.0001) | Yes (0.0020) | Yes (<0.0001) | Yes (<0.0001) |
| **2500 vs 10,000** | Yes (<0.0001) | Yes (<0.0001) | Yes (<0.0001) | Yes (<0.0001) |
| **2500 vs 12,500** | Yes (<0.0001) | Yes (<0.0001) | Yes (<0.0001) | Yes (<0.0001) |
| **2500 vs 15,000** | Yes (<0.0001) | Yes (<0.0001) | Yes (<0.0001) | Yes (<0.0001) |
| **5000 vs 7500** | Yes (0.0099) | No (0.3604) | Yes (0.0367) | No (0.1719) |
| **5000 vs 10,000** | Yes (<0.0001) | Yes (0.0224) | Yes (<0.0001) | Yes (0.0018) |
| **5000 vs 12,500** | Yes (<0.0001) | Yes (<0.0001) | Yes (<0.0001) | Yes (<0.0001) |
| **5000 vs 15,000** | Yes (<0.0001) | Yes (<0.0001) | Yes (<0.0001) | Yes (<0.0001) |
| **7500 vs 10,000** | No (0.3554) | No (0.7913) | No (0.0669) | No (0.5161) |
| **7500 vs 12,500** | Yes (<0.0001) | Yes (0.0169) | Yes (<0.0001) | Yes (<0.0001) |
| **7500 vs 15,000** | Yes (<0.0001) | Yes (0.0003) | Yes (<0.0001) | Yes (<0.0001) |
| **10,000 vs 12,500** | No (0.0608) | No (0.3054) | Yes (0.0064) | Yes (0.0156) |
| **10,000 vs 15,000** | Yes (0.0008) | Yes (0.0159) | Yes (<0.0001) | Yes (<0.0001) |
| **12,500 vs 15,000** | No (0.7558) | No (0.7788) | Yes (0.0116) | No (0.4456) |

***Supplementary Table 2:*** *Summary of Tukey’s multiple comparisons test on LDH lysate, CTG-3D, volume, and luminescence day 7 measurements of NCI-H1650 and NCI-H1650.LMC spheroids.*

| **Day 7** | **Significant (p-value)** | | | |
| --- | --- | --- | --- | --- |
|  | **LDH Lysate** | **CTG-3D** | **Volume** | **Luminescence** |
| **2500 vs 5000** | Yes (<0.0001) | No (0.0735) | Yes (<0.0001) | No (0.2137) |
| **2500 vs 7500** | Yes (<0.0001) | Yes (<0.0001) | Yes (<0.0001) | Yes (0.0036) |
| **2500 vs 10,000** | Yes (<0.0001) | Yes (<0.0001) | Yes (<0.0001) | Yes (0.0002) |
| **2500 vs 12,500** | Yes (<0.0001) | Yes (<0.0001) | Yes (<0.0001) | Yes (<0.0001) |
| **2500 vs 15,000** | Yes (<0.0001) | Yes (<0.0001) | Yes (<0.0001) | Yes (<0.0001) |
| **5000 vs 7500** | No (0.2532) | Yes (0.0002) | Yes (<0.0001) | No (0.5884) |
| **5000 vs 10,000** | No (0.1557) | Yes (<0.0001) | Yes (<0.0001) | No (0.1365) |
| **5000 vs 12,500** | Yes (0.0104) | Yes (<0.0001) | Yes (<0.0001) | Yes (0.0202) |
| **5000 vs 15,000** | Yes (0.0152) | Yes (<0.0001) | Yes (<0.0001) | Yes (0.0003) |
| **7500 vs 10,000** | No (0.9999) | No (0.0740) | Yes (<0.0001) | No (0.9433) |
| **7500 vs 12,500** | No (0.8080) | No (0.0802) | Yes (<0.0001) | No (0.5401) |
| **7500 vs 15,000** | No (0.8657) | Yes (0.0002) | Yes (<0.0001) | Yes (0.0402) |
| **10,000 vs 12,500** | No (0.9129) | No (>0.9999) | Yes (<0.0001) | No (0.9675) |
| **10,000 vs 15,000** | No (0.9478) | No (0.3729) | Yes (<0.0001) | No (0.2800) |
| **12,500 vs 15,000** | No (>0.9999) | No (0.3536) | Yes (<0.0001) | No (0.7512) |

**Supplementary Table 3:** Summary of Sidak’s multiple comparisons test on LDH lysate, CTG-3D, volume, and luminescence day 0 vs day 7 measurements of NCI-H1650 and NCI-H1650.LMC spheroids.

| **Day 0 vs Day 7** | **Significant (p-value)** | | | | | |
| --- | --- | --- | --- | --- | --- | --- |
|  | **LDH Lysate** | | **CTG-3D** | | **Volume** | **Luminescence** |
| **2500** | | Yes (<0.0001) | | Yes (<0.0001) | Yes (<0.0001) | Yes (<0.0001) |
| **5000** | | Yes (<0.0001) | | Yes (<0.0001) | Yes (<0.0001) | Yes (<0.0001) |
| **7500** | | Yes (<0.0001) | | Yes (<0.0001) | Yes (<0.0001) | Yes (0.0001) |
| **10,000** | | Yes (<0.0001) | | Yes (<0.0001) | Yes (<0.0001) | Yes (0.0021) |
| **12,500** | | Yes (<0.0001) | | Yes (<0.0001) | Yes (<0.0001) | No (0.7853) |
| **15,000** | | Yes (<0.0001) | | Yes (<0.0001) | Yes (<0.0001) | No (0.9767) |

**Supplementary Table 4:** Summary of Tukey’s multiple comparisons test on whole NCI-H1650 spheroids analyzed via MTS, PrestoBlue, LDH, and CTG-measurements.

| **Whole** | **Significant (p-value)** | | | |
| --- | --- | --- | --- | --- |
|  | **MTS** | **PrestoBlue** | **LDH** | **CTG-3D** |
| **2500 vs 5000** | No (0.9619) | No (0.5998) | Yes (<0.0001) | Yes (<0.0001) |
| **2500 vs 7500** | No (0.5451) | No (0.1885) | Yes (<0.0001) | Yes (<0.0001) |
| **2500 vs 10,000** | Yes (0.0285) | Yes (0.0395) | Yes (<0.0001) | Yes (<0.0001) |
| **2500 vs 12,500** | Yes (<0.0001) | Yes (0.0004) | Yes (<0.0001) | Yes (<0.0001) |
| **2500 vs 15,000** | Yes (<0.0001) | Yes (<0.0001) | Yes (<0.0001) | Yes (<0.0001) |
| **5000 vs 7500** | No (0.9524) | No (0.9724) | Yes (<0.0001) | Yes (0.0003) |
| **5000 vs 10,000** | No (0.1882) | No (0.6918) | Yes (<0.0001) | Yes (<0.0001) |
| **5000 vs 12,500** | Yes (0.0006) | Yes (0.0435) | Yes (<0.0001) | Yes (<0.0001) |
| **5000 vs 15,000** | Yes (0.0002) | Yes (0.0005) | Yes (<0.0001) | Yes (<0.0001) |
| **7500 vs 10,000** | No (0.6657) | No (0.9816) | Yes (<0.0001) | Yes (0.0403) |
| **7500 vs 12,500** | Yes (0.0092) | No (0.2301) | Yes (<0.0001) | Yes (<0.0001) |
| **7500 vs 15,000** | Yes (0.0038) | Yes (0.0050) | Yes (<0.0001) | Yes (<0.0001) |
| **10,000 vs 12,500** | No (0.3033) | No (0.6245) | Yes (<0.0001) | Yes (0.0001) |
| **10,000 vs 15,000** | No (0.1767) | Yes (0.0343) | Yes (<0.0001) | Yes (<0.0001) |
| **12,500 vs 15,000** | No (0.9997) | No (0.6330) | Yes (<0.0001) | Yes (0.0350) |

**Supplementary Table 5:** Summary of Tukey’s multiple comparisons test on dissociated NCI-H1650 spheroids analyzed via MTS, PrestoBlue, LDH, and CTG-measurements.

| **Dissociated** | **Significant (p-value)** | | | |
| --- | --- | --- | --- | --- |
|  | **MTS** | **PrestoBlue** | **LDH** | **CTG-3D** |
| **2500 vs 5000** | No (0.7356) | Yes (0.0001) | Yes (<0.0001) | Yes (<0.0001) |
| **2500 vs 7500** | Yes (0.0225) | Yes (<0.0001) | Yes (<0.0001) | Yes (<0.0001) |
| **2500 vs 10,000** | Yes (0.0002) | Yes (<0.0001) | Yes (<0.0001) | Yes (<0.0001) |
| **2500 vs 12,500** | Yes (<0.0001) | Yes (<0.0001) | Yes (<0.0001) | Yes (<0.0001) |
| **2500 vs 15,000** | Yes (<0.0001) | Yes (<0.0001) | Yes (<0.0001) | Yes (<0.0001) |
| **5000 vs 7500** | No (0.4185) | No (0.6459) | Yes (<0.0001) | Yes (<0.0001) |
| **5000 vs 10,000** | Yes (0.0119) | Yes (0.0001) | Yes (<0.0001) | Yes (<0.0001) |
| **5000 vs 12,500** | Yes (<0.0001) | Yes (<0.0001) | Yes (<0.0001) | Yes (<0.0001) |
| **5000 vs 15,000** | Yes (<0.0001) | Yes (<0.0001) | Yes (<0.0001) | Yes (<0.0001) |
| **7500 vs 10,000** | No (0.5913) | Yes (0.0138) | Yes (<0.0001) | Yes (0.0003) |
| **7500 vs 12,500** | Yes (0.0003) | Yes (<0.0001) | Yes (<0.0001) | Yes (<0.0001) |
| **7500 vs 15,000** | Yes (<0.0001) | Yes (<0.0001) | Yes (<0.0001) | Yes (<0.0001) |
| **10,000 vs 12,500** | Yes (0.0392) | No (0.1109) | Yes (<0.0001) | Yes (<0.0001) |
| **10,000 vs 15,000** | Yes (<0.0001) | No (0.1384) | Yes (<0.0001) | Yes (<0.0001) |
| **12,500 vs 15,000** | No (0.1120) | No (>0.9999) | Yes (<0.0001) | Yes (<0.0001) |

**Supplementary Table 6:** Summary of Sidak’s multiple comparisons test on MTS, PrestoBlue, LDH, and CTG-3D measurements on whole vs dissociated NCI-H1650 spheroids.

| **Whole vs dissociated** | **Significant (p-value)** | | | |
| --- | --- | --- | --- | --- |
|  | **MTS** | **PrestoBlue** | **LDH** | **CTG-3D** |
| **2500** | No (0.9936) | Yes (0.0032) | No (0.8300) | No (>0.9999) |
| **5000** | No (0.8379) | Yes (<0.0001) | No (0.5373) | No (0.6584) |
| **7500** | No (0.1956) | Yes (<0.0001) | No (0.9996) | Yes (0.0020) |
| **10,000** | No (0.1521) | Yes (<0.0001) | No (0.6332) | Yes (<0.0001) |
| **12,500** | Yes (0.0129) | Yes (<0.0001) | Yes (0.0038) | Yes (<0.0001) |
| **15,000** | Yes (<0.0001) | Yes (<0.0001) | Yes (0.0070) | Yes (<0.0001) |

**Supplementary Table 7:** Patient description.

| **OvC case** | **Diagnosis** | **Age** | **FIGO** | **Chemotherapy** | **Follow-up** | **Patient Status** |
| --- | --- | --- | --- | --- | --- | --- |
| **1** | HGSC | 70 | IVB | Adjuvant | 16 months | Alive without evidence of disease |
| **2** | LGSC | 40 | IIIB | Neoadjuvant | 3 months | Alive without evidence of disease |
| **3** | HGSC | 73 | IIB | No (intolerance) | 19 months | Died of disease |
| **4** | HGSC | 77 | IIIC | Adjuvant | 18 months | Alive with disease |
| **5** | HGSC | 81 | IIIC | Adjuvant | 22 months | Alive with disease |
| **6** | Endometriod Carcinoma | 65 | IC | Adjuvant | 20 months | Alive without evidence of disease |
| **7** | Adult granulosa cell tumor | 85 | IA | No | 0 months | Died of other causes |
| **8** | HGSC | 62 | IIIC | Adjuvant | - | Lost for follow-up |
| **9** | Serous Borderline Tumor | 25 | IA | No | 26 months | Alive without evidence of disease |
| **10** | Endometriod Carcinoma | 50 | IC | Adjuvant | 7 months | Alive without evidence of disease |
| **11** | HGSC | 60 | IC | Adjuvant | 10 months | Alive without evidence of disease |
| **12** | LGSC | 85 | IIIB | Adjuvant | 6 months | Died of disease |
| **13** | Mucinous Borderline Tumor | 84 | IA | No | 11 months | Alive without evidence of disease |
| **14** | HGSC | 62 | IIIC | Neodjuvant+ adjuvant | 14 months | Alive with disease |
| **15** | HGSC | 51 | IIIC | Neodjuvant+ adjuvant | 12 months | Alive without evidence of disease |
| **16** | HGSC | 44 | IIIC | Adjuvant | 10 months | Alive with disease |

HGSC: High-grade Serous Carcinoma; LGSC: Low-grade Serous Carcinoma.

**Supplementary Table 8:** Clinical Pathological data of OvC patients.

| **Age** |  |  |
| --- | --- | --- |
| **Medium age (years)** | 63 |  |
| **Age range** | 25-85 |  |
|  | **Number of cases (N = 16)** | **Percentage (%)** |
| **<60** | 5 | 31 |
| **≥60** | 11 | 69 |
| **Histopathology diagnosis** | **Number of cases (N = 16)** | **Percentage (%)** |
| **High-Grade Serous carcinoma*** | 9 | 56.3 |
| **Low-Grade Serous carcinoma**** | 2 | 12.5 |
| **Serous Borderline Tumor** | 1 | 6.3 |
| **Endometriod Carcinoma** | 2 | 12.5 |
| **Mucinous borderline tumor** | 1 | 6.3 |
| **Adult Granulosa Cell Tumor** | 1 | 6.3 |
| **FIGO staging** | **Number of cases (N = 16)** | **Percentage (%)** |
| **Stage I** | 6 | 38 |
| **Stage II** | 1 | 6 |
| **Stage III** | 8 | 50 |
| **Stage IV** | 1 | 6 |

*2 and **1 case with chemotherapy treatment before surgery.

**Supplementary Table 9:** Summary of proliferation (ki67+ cells) and apoptosis (cleaved caspase 3+ cells) level quantified by immunohistochemistry analysis.

| **OvC case** | **Marker** | **Tumor Sample** | **OvC-PDE (Day 21)** |
| --- | --- | --- | --- |
| **2** | Ki67+ cells (%) | 50% | 25-50% |
|  | Cleaved Caspase 3+ cells (%) | 5-25% | 5-25% |
| **3** | Ki67+ cells (%) | 50-75% | 1-5% |
|  | Cleaved Caspase 3+ cells (%) | 50-75% | 50-75% |
| **4** | Ki67+ cells (%) | >75% | 50-75% |
|  | Cleaved Caspase 3+ cells (%) | 5-25% | 50-75% |
| **5** | Ki67+ cells (%) | 25-50% | 5-25% |
|  | Cleaved Caspase 3+ cells (%) | 25-50% | 1-5% |

**Supplementary Table 10:** Drug-induced cell death evaluation of OvC-PDE on proliferation and apoptosis levels by case.

| **OvC case** | **Ki67+ cells (%)** | | | | **Casp3+ cells (%)** | | | |
| --- | --- | --- | --- | --- | --- | --- | --- | --- |
|  | **Untreated** | **C** | **P** | **C+P** | **Untreated** | **C** | **P** | **C+P** |
| **2** | 25-50 | 25-50 | 50 | 25-50 | 5-25 | 5-25 | 5-25 | 5-25 |
| **3** | 1-5 |  |  | <1 | 50-75 |  |  | 25-50 |
| **4** | 50-75 | <1 | <1 | <1 | 50-75 | 5-25 | 5-25 | 25-50 |
| **5** | 5-25 | <1 | NA | NA | 1-5 | <1 | NA | NA |
| **7** | <1 | NA | NA | <1 | 25-50 | NA | 25-50 | 50-75 |
| **8** | 50-75 | 5-25 | NA | No viable sample | >75 | >75 | NA | No viable sample |
| **9** | <1 | <1 | <1 | <1 | 1-5 | 5-25 | 50 | 5-25 |
| **10** | 1 | NA | >75 | NA | 1 | NA | <1 | NA |
| **11** | 5-25 | NA | NA | NA | <1 | NA | NA | NA |
| **12** | 1-5 | 1-5 | <1 | NA | 5-25 | >75 | 50-75 | NA |
| **13** | 1-5 | NA | <1 | <1 | 1-5 | >75 | 5-25 | >75 |
| **14** | <1 | <1 | <1 | NA | <1 | <1 | <1 | NA |
| **15** | <1 | <1 | NA | NA | 5-25 | <1 | NA | NA |
| **16** | 50-75 | NA | <1 | NA | 25-50 | NA | <1 | NA |
